# Mimicking Elementary Reactions of Manganese Lipoxygenase Using Mn-hydroxo and Mn-alkylperoxo Complexes

**DOI:** 10.3390/molecules26237151

**Published:** 2021-11-25

**Authors:** Adedamola A. Opalade, Elizabeth N. Grotemeyer, Timothy A. Jackson

**Affiliations:** Department of Chemistry and Center for Environmentally Beneficial Catalysis, The University of Kansas, 1567 Irving Hill Road, Lawrence, KS 66045, USA; aaopalade@ku.edu (A.A.O.); egrotemeyer@ku.edu (E.N.G.)

**Keywords:** manganese enzymes, lipoxygenase, hydrogen-atom transfer, ligand substitution, alkylperoxo

## Abstract

Manganese lipoxygenase (MnLOX) is an enzyme that converts polyunsaturated fatty acids to alkyl hydroperoxides. In proposed mechanisms for this enzyme, the transfer of a hydrogen atom from a substrate C-H bond to an active-site Mn^III^-hydroxo center initiates substrate oxidation. In some proposed mechanisms, the active-site Mn^III^-hydroxo complex is regenerated by the reaction of a Mn^III^-alkylperoxo intermediate with water by a ligand substitution reaction. In a recent study, we described a pair of Mn^III^-hydroxo and Mn^III^-alkylperoxo complexes supported by the same amide-containing pentadentate ligand (^6Me^dpaq). In this present work, we describe the reaction of the Mn^III^-hydroxo unit in C-H and O-H bond oxidation processes, thus mimicking one of the elementary reactions of the MnLOX enzyme. An analysis of kinetic data shows that the Mn^III^-hydroxo complex [Mn^III^(OH)(^6Me^dpaq)]^+^ oxidizes TEMPOH (2,2′-6,6′-tetramethylpiperidine-1-ol) faster than the majority of previously reported Mn^III^-hydroxo complexes. Using a combination of cyclic voltammetry and electronic structure computations, we demonstrate that the weak Mn^III^-N(pyridine) bonds lead to a higher Mn^III/II^ reduction potential, increasing the driving force for substrate oxidation reactions and accounting for the faster reaction rate. In addition, we demonstrate that the Mn^III^-alkylperoxo complex [Mn^III^(OO*^t^*Bu)(^6Me^dpaq)]^+^ reacts with water to obtain the corresponding Mn^III^-hydroxo species, thus mimicking the ligand substitution step proposed for MnLOX.

## 1. Introduction

Manganese lipoxygenase (MnLOX) is an enzyme that oxidizes C-H bonds of polyunsaturated fatty acids to generate alkyl hydroperoxide products [1,2,3,4]. The hydroperoxides are metabolized to oxylipins, such as leukotrienes and jasmonates, which act as inflammatory mediators and reproductive or growth regulators in plants [5,6]. MnLOXs are also found in fungi that are pathogenic to crops such as wheat (*Gaeumannomyces graminis*) [3] and rice (*Magnaporthe oryzae*) [7,8]. Because of the role of these pathogens in crop disease, MnLOXs have garnered interest as a target for pathogenesis [9].

**Scheme 1:**
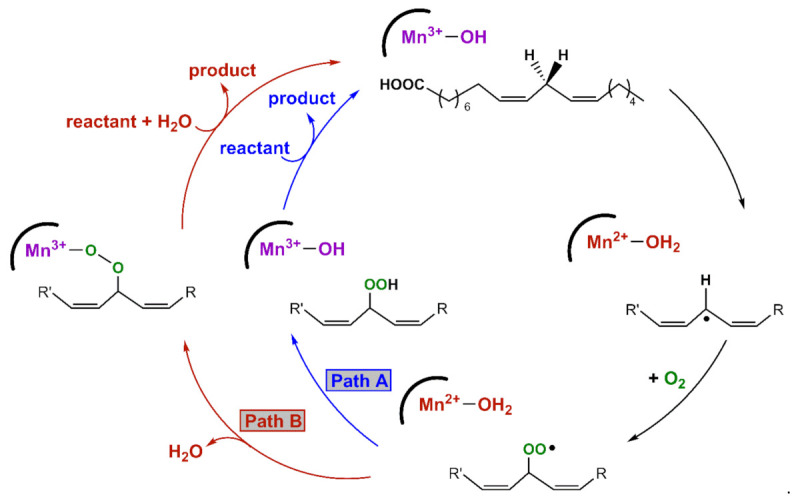
Proposed mechanisms for MnLOX.

An X-ray crystal structure of the MnLOX enzyme from the fungus *Magnaporthe oryzae* provides important information regarding the structure of the enzyme [10]. The active site consists of a mononuclear Mn center coordinated by three histidine ligands, a carboxylate from the C-terminus of the protein, a carbonyl donor from an asparagine group, and a solvent molecule. The crystals contained a Mn^II^ center, and the solvent ligand was presumed to be water. It is commonly assumed that the Mn^III^ oxidation state contains a hydroxo ligand [9]. Kinetic studies of MnLOX enzymes [4,8,9,11] and comparisons to Fe-dependent LOX enzymes [12,13] have led to the proposed mechanism in Scheme 1. The rate-determining step for substrate oxidation involves the abstraction of a hydrogen atom from the fatty acid substrate by an active-site Mn^III^-hydroxo unit, yielding a carbon-centered radical and a Mn^II^-aqua species. In MnLOX from *M. oryzae*, the *k*_cat_ parameter shows a large substrate C-H/C-D kinetic isotope effect of 60–80 [9], consistent with dominant hydrogen-atom tunneling in the rate-determining step [14]. The carbon-based substrate radical can rearrange, after which it is trapped by O_2_ to generate an oxygen-centered radical. This radical can abstract a hydrogen-atom from the Mn^II^-aqua complex to yield product and regenerate the Mn^III^-hydroxo center (Path A in Scheme 1). Alternatively, the oxygen-centered radical can displace the aqua ligand and oxidize the Mn^II^ center to yield a Mn^III^-alkylperoxo complex (Path B in Scheme 1). Support for this alternate pathway is provided by an X-ray crystal structure of an Fe-alkylperoxo complex from soybean lipoxygenase [15]. In this path, the Mn^III^-alkylperoxo complex reacts with water via a ligand substitution reaction to yield the substrate and the Mn^III^-hydroxo center.

The proposed mechanism for MnLOX has inspired attempts to model the rate-determining hydrogen-atom abstraction step, where a C-H bond transfers a hydrogen-atom to the Mn^III^-hydroxo center. Stack and co-workers were the first to report a Mn^III^-hydroxo center capable of oxidizing a C-H bond [16]. Using the neutral, pentadentate PY5 ligand, the Mn^III^-hydroxo complex [Mn^III^(PY5)(OH)]^2+^ (PY5 = 2,6-bis(bis(2-pyridyl)-methoxymethane)pyridine) is able to oxidize several hydrocarbons, including xanthene [16]. The bis-benzylic C-H bonds in xanthene provide a reasonable mimic of the bis-allylic C-H bonds in the native substrate of MnLOX. More recently, our lab reported xanthene oxidation by a pair of Mn^III^-hydroxo complexes supported by anionic, pentadentate ligands, [Mn^III^(OH)(dpaq)]^+^ and [Mn^III^(OH)(dpaq^2Me^)]^+^ (dpaq = 2-[bis(pyridin-2ylmethyl)]amino-*N*-quinolin-8-yl-acetamidate) [17,18]. An analysis of kinetic data for these complexes revealed that the rate of xanthene oxidation by [Mn^III^(PY5)(OH)]^2+^ is roughly 10 and 30 fold faster than that of [Mn^III^(OH)(dpaq)]^+^ and [Mn^III^(OH)(dpaq^2Me^)]^+^, respectively [16,18,19,20].

An alternative means of comparing reactivity of Mn^III^-hydroxo complexes has been achieved using TEMPOH (1-hydroxy-2,2,6,6-tetramethyl-piperidine) [20,21]. While TEMPOH does not model the MnLOX substrate, its relatively weak O-H bond (BDFE = 66.5 kcal mol^−1^ in MeCN at 298 K; BDFE = bond dissociation free energy) [22] permits detailed kinetic studies for metal complexes with a range of oxidative capabilities because of the combination of a weak O-H bond, poor Brøonsted acidity (p*K*_a_ = 41 in MeCN), and the difficulty of oxidation (TEMPOH^+/∙^ *E*_p,a_ = 0.71 V vs. Fc^+/0^) [23]. TEMPOH typically reacts by a concerted proton-electron transfer (CPET) step, yielding the stable TEMPO radical as the sole product. Kovacs et al. reported a thiolate-ligated Mn^III^-hydroxo complex, [Mn^III^(OH)(S^Me2^N_4_(tren))]^+^, that oxidizes TEMPOH with a very fast second-order rate constant (*k*_2_) of 2.1 × 10^3^ M^−1 ^s^−1^ at 25 °C [24]. For comparison, the [Mn^III^(OH)(dpaq)]^+^ and [Mn^III^(OH)(dpaq^2Me^)]^+^ complexes also oxidize TEMPOH, but rate data are available at much lower temperatures (*k*_2_ = 1.1 and 3.9 M^−1^ s^−1^, respectively, at −35 °C) [17]. Derivatives of [Mn^III^(OH)(dpaq)]^+^, with electron-withdrawing and electron-donating groups appended para to the amidate function of dpaq, showed rate enhancements in TEMPOH oxidation for electron-withdrawing substituents [20]. More recently, a Mn^III^-hydroxo complex with an intramolecular hydrogen bond was also shown to exhibit more rapid rates for TEMPOH oxidation than an analogue without the hydrogen bond [25].

**Scheme 2:**
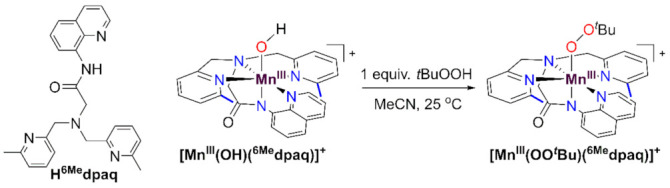
Reaction of the Mn^III^-hydroxo complex [Mn^III^(OH)(^6Me^dpaq)]^+^ with *^t^*BuOOH to generate the Mn^III^-alkylperoxo complex [Mn^III^(OO*^t^*Bu)(^6Me^dpaq)]^+^. The structure of the protonated Hdpaq^6Me^ ligand is shown on the far left.

While there are now several studies mimicking the C-H bond oxidation step of MnLOX, there are far fewer examples investigating the potential ligand substitution reaction of the Mn^III^-alkylperoxo intermediate. Kovacs and co-workers have described a family of Mn^III^-alkylperoxo complexes supported by anionic, pentadentate N_4_S^−^ and N_4_O^−^ ligands [26,27,28]. These complexes decay thermally by O-O homolysis, which is distinct from the chemistry proposed for the Mn^III^-alkylperoxo complex of MnLOX (Scheme 1). Our lab reported Mn^III^-alkylperoxo complexes supported by the dpaq and dpaq^2Me^ ligands; however, the instability of these complexes, and the requirement of a large excess of *^t^*BuOOH to achieve their formation, made studies of reactivity unfeasible [29]. More recently, we reported that a derivative of the dpaq ligand, with 6-Me-pyridyl groups, is able to support Mn^III^-alkylperoxo complexes that are stable at room temperature and can be formed by adding an equivalent of an alkylhydroperoxide to the corresponding Mn^III^-hydroxo complex [Mn^III^(OH)(^6Me^dpaq)]^+^ (Scheme 2) [30]. The observation that a set of thermally stable Mn^III^-hydroxo and Mn^III^-alkylperoxo complexes could be generated using the same supporting ligand presents a unique opportunity to model multiple steps in the proposed mechanism of MnLOX.

In this present study, we describe the reactivity of [Mn^III^(OH)(^6Me^dpaq)]^+^ towards TEMPOH, thereby mimicking the initial substrate oxidation step in MnLOX. To understand the reason behind rate variations among the Mn^III^-hydroxo complexes, we explore the spectroscopic and thermodynamic properties of [Mn^III^(OH)(^6Me^dpaq)]^+^ using the density functional theory (DFT) computations. We also examine the reaction of the Mn^III^-alkylperoxo complex [Mn^III^(OO*^t^*Bu)(^6Me^dpaq)]^+^ with water. This reaction yields the Mn^III^-hydroxo complex [Mn^III^(OH)(^6Me^dpaq)]^+^, thereby mimicking a potential step for substrate release by MnLOX.

## 2. Materials and Methods

All chemicals obtained from commercial sources were of ACS grade or higher and were used as obtained, unless otherwise noted. Acetonitrile, diethyl ether, and methanol were dried and degassed using a PureSolv Micro solvent purification system. The H^6Me^dpaq ligand and the [Mn^II^(OH_2_)(^6Me^dpaq)](OTf), [Mn^III^(OH)(^6Me^dpaq)](OTf), and [Mn^III^(OO^t^Bu)(^6Me^dpaq)](OTf) complexes were synthesized according to a previously reported procedure (OTf^−^ = trifluoromethanesulfonate) [30]. All synthetic experiments were performed under dinitrogen atmosphere in a glovebox unless otherwise noted. Electronic absorption experiments were performed using either a Varian Cary 50 Bio UV-visible spectrophotometer (Agilent Technologies, Santa Clara, CA, USA), equipped with a Unisoku cryostat and stirrer (for low temperature experiments) or a Quantum Northwest temperature controller equipped with a stirrer (for high temperature experiments). Electrospray ionization mass spectrometry (ESI-MS) experiments were performed using an LCT Premier MicroMass electrospray time-of-flight instrument (Waters, Milford, MA, USA).

### 2.1. Kinetic Studies of TEMPOH and Xanthene Oxidation by [Mn^III^(OH)(^6Me^dpaq)](OTf)

A 1.25 mM solution of [Mn^III^(OH)(^6Me^dpaq)]^+^ was prepared in 2.0 mL of MeCN in a nitrogen-filled glovebox and transferred to a quartz cuvette that was sealed with a rubber septum. The cuvette was removed from the glovebox and allowed to equilibrate at –35 °C for 10 min on the UV-vis spectrometer. A 100 μL solution of TEMPOH, with concentrations ranging from 10–60 equiv. relative to [Mn^III^(OH)(^6Me^dpaq)]^+^, was added to the cuvette using a gastight syringe that had been purged with N_2_ gas. The addition of TEMPOH led to the disappearance of the 510 nm electronic absorption feature of [Mn^III^(OH)(^6Me^dpaq)]^+^. The change in absorbance as a function of time was fit to obtain a pseudo-first-order rate constant (*k_obs_*). The reported *k_obs_* represent an average from three separate measurements. A linear fit of the plot of *k_obs_* vs. the concentration of TEMPOH provided the second-order rate constant (*k*_2_).

The reactivity of [Mn^III^(OH)(^6Me^dpaq)]^+^ with xanthene was investigated using a similar approach. In this case, 250 equiv. xanthene (relative to Mn) was prepared anaerobically in 300 μL dichloromethane in a 400 mL vial. A solution of xanthene was added to 2 mL of a 1.25 mL solution of [Mn^III^(OH)(^6Me^dpaq)]^+^ that had equilibrated at 50 °C for 10 min on the spectrometer. The decay of the 510 nm feature of the [Mn^III^(OH)(^6Me^dpaq)]^+^ was monitored by electronic absorption spectroscopy over a period of 1000 min.

### 2.2. Reaction of [Mn^III^(OO^t^Bu)(^6Me^dpaq)]^+^ with Protic Solvents and Kinetic Investigations with Water

The propensity for [Mn^III^(OO*^t^*Bu)(^6Me^dpaq)]^+^ to ligand substitution reactions was first discovered in an attempt to prepare the complex in various protic solvents. In a representative procedure, [Mn^III^(OO*^t^*Bu)(^6Me^dpaq)]^+^ was prepared in MeCN and then dried in vacuo to remove the solvent, leaving behind an oily film. The oily film was taken into the glovebox and dissolved in 2,2,2-trifluoroethanol (TFE). The solution was transferred to a quartz cuvette, sealed with a rubber septum, and wrapped with Parafilm. The sample was then removed from the glovebox, inserted in the UV-vis spectrometer, and heated to 50 °C. The initial absorption spectra collected under these conditions revealed an electronic absorption band of [Mn^III^(OO*^t^*Bu)(^6Me^dpaq)]^+^ at 650 nm. This band decayed over the course of 60 min, with the concomitant formation of a band at 510 nm (Figure S1). An ESI-MS analysis of the product solution revealed a prominent ion peak at 564.07 *m*/*z*, consistent with [Mn^III^(OCH_2_CF_3_)(^6Me^dpaq)]^+^ (*m*/*z* = 564.14; see Figure S2). Thus, while [Mn^III^(OO*^t^*Bu)(^6Me^dpaq)]^+^ initially forms through the dissolution of the [Mn^III^(OO*^t^*Bu)(^6Me^dpaq)](OTf) salt in TFE, a ligand substitution reaction occurs that replaces the alkylperoxo ligand with a CF_3_CH_2_O^-^ ligand. This ligand substitution reaction with TFE was only observed at 50 °C. A similar, albeit far more rapid, ligand substitution reaction occurs via the dissolution of [Mn^III^(OO*^t^*Bu)(^6Me^dpaq)](OTf) in MeOH at 25 °C (Figure S3). Moreover, the addition of 100 μL MeOH to a 1.0 mM solution of [Mn^III^(OO*^t^*Bu)(^6Me^dpaq)](OTf) in MeCN leads to its conversion to the corresponding Mn^III^-methoxy complex (Figures S3 and S4).

To determine rate constants for ligand substitution reactions of [Mn^III^(OO*^t^*Bu)(^6Me^dpaq)]^+^ with water, we prepared an acetonitrile solution of [Mn^III^(OO*^t^*Bu)(^6Me^dpaq)]^+^ in a glovebox, transferred the solution to a quartz cuvette sealed with a pierceable rubber septum and wrapped with Parafilm. The cuvette was removed from the glovebox and an aliquot of degassed H_2_O (100–400 equiv. relative to [Mn^III^(OO*^t^*Bu)(^6Me^dpaq)]^+^) was injected while monitoring the reaction by electronic absorption spectroscopy at 25 °C.

### 2.3. Cyclic Voltammetry

Cyclic voltammograms were recorded using a Basi^®^ PalmSens EmStat3+ potentiostat (PalmSens BV, Houten, Utrecht, The Netherlands). The working electrode was a glassy carbon electrode with a Pt wire as the counter electrode. A 0.01 M AgCl solution was prepared using a 0.1 M Bu_4_NPF_6_ electrolyte solution in CH_3_CN. The 0.01 M AgCl solution was used for the Ag/AgCl quasi-reference electrode. The Fc^+^/Fc potential was measured as an external reference. Additionally, 2 mM solutions of [Mn^III^(OH)(^6Me^dpaq)](OTf) were prepared from 10 mL of a degassed 0.1 M Bu_4_NPF_6_ electrolyte solution in CH_3_CN. These sample solutions were sparged with nitrogen gas with the aid of Teflon tubing for 15 min before measurement. The Teflon tubing was placed above the surface of the solution during measurement to continue flushing the headspace, without disturbing the solution in the electrochemical cell. All measurements were performed at room temperature. Data were referenced to the cathodic peak potential for Fc^+^/Fc in MeCN.

### 2.4. Electronic Structure Calculations

All DFT calculations were performed using ORCA 4.2.1. [31]. For the geometry optimizations the B3LYP [32,33] functional with the def2-TZVP basis set for Mn, N and O atoms were used, while the def2-SVP basis set was used for C and H atoms [34,35]. Grimme’s D3 dispersion correction [36,37,38,39] was also applied with a fine integration grid (Grid6 and GridX6 in ORCA). Analytical frequency calculations were performed using the same level of theory. The zero-point energies, thermal corrections, and entropies were obtained from the analytical frequency calculations. Single point energies were obtained for all structures using the same B3LYP-D3 functional but with the larger def2-TZVPP basis set on all atoms and a finer integration grid (Grid7 and GridX7). In all cases, solvation was accounted for by using the SMD solvation model with default parameters for acetonitrile [40]. The RIJCOSX approximation, together with def2/J auxiliary basis set, was used for all calculations [41,42]. A detailed discussion of the calculation of thermodynamic parameters is included in the Supplementary Materials.

## 3. Results and Discussion

### 3.1. Formation of [Mn^III^(OH)(^6Me^dpaq)]^+^ by Aerobic Oxidation

**Figure 1:**
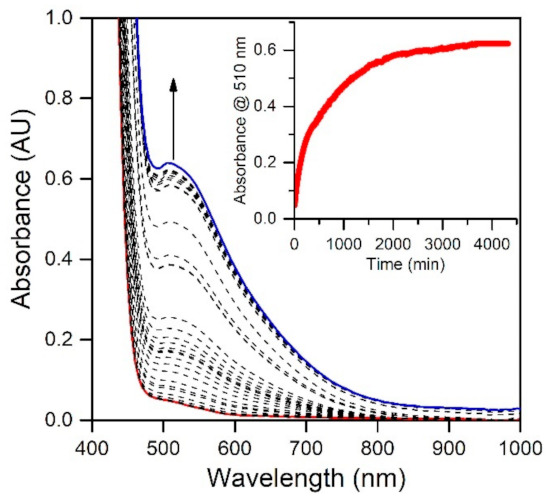
Electronic absorption spectra showing the reaction of a 2.5 mM solution of [Mn^II^(H_2_O)(^6Me^dpaq)](OTf) in CH_3_CN (red trace) with dioxygen at 25 °C. The dashed traces show the reaction progress; the blue trace represents the final spectrum. The inset shows the growth in absorbance at 510 nm over time.

In a previous study, we reported that the Mn^III^-hydroxo complex [Mn^III^(OH)(^6Me^dpaq)]^+^ could be generated via the oxidation of the Mn^II^-aqua complex [Mn^II^(H_2_O)(^6Me^dpaq)](OTf) using 0.5 equiv. iodosobenzene (PhIO) [30]. Because a number of Mn^III^-hydroxo complexes can be generated by aerobic oxidation of their Mn^II^ analogues [17,18,20,24,43,44,45], we explored the reaction of [Mn^II^(H_2_O)(^6Me^dpaq)](OTf) with O_2_. When dissolved in MeCN, the [Mn^II^(H_2_O)(^6Me^dpaq)](OTf) complex shows a weak electronic absorption shoulder, stretching from approximately 600 to 490 nm (Figure 1). The exposure of a MeCN solution of this complex to O_2_ results in the eventual growth of a more intense band near 510 nm that is attributed to [Mn^III^(OH)(^6Me^dpaq)]^+^ [30]. This reaction is very slow, with approximately 75% conversion to the Mn^III^-hydroxo complex achieved after 48 h (Figure 1). In contrast, the [Mn^II^(dpaq)](OTf) complex and several derivatives show complete oxidation to Mn^III^ products within 0.5–5 h [17,18,20]. The sluggish nature of the reaction of [Mn^II^(H_2_O)(^6Me^dpaq)]^+^ with O_2_ may be caused by a more electron-deficient Mn^II^ center. The 6-Me-pyridyl groups in [Mn^II^(H_2_O)(^6Me^dpaq)]^+^ give rise to Mn-N_pyridine_ bonds that are 0.02–0.04 Å longer than [Mn^II^(dpaq)](OTf) and its derivatives [17,18]. These longer bonds presumably mitigate electron donation from the pyridyl ligands to the Mn^II^ center, leading to a more electron-poor metal center. Similarly, the [Mn^II^(dpaq^5NO2^)](OTf) complex, which contains a strongly electron-donating nitro group on the quinolinyl moiety, showed essentially no reactivity with dioxygen [20].

### 3.2. Spectroscopic Properties and Electronic Structure of [Mn^III^(OH)(^6Me^dpaq)]^+^

**Figure 2:**
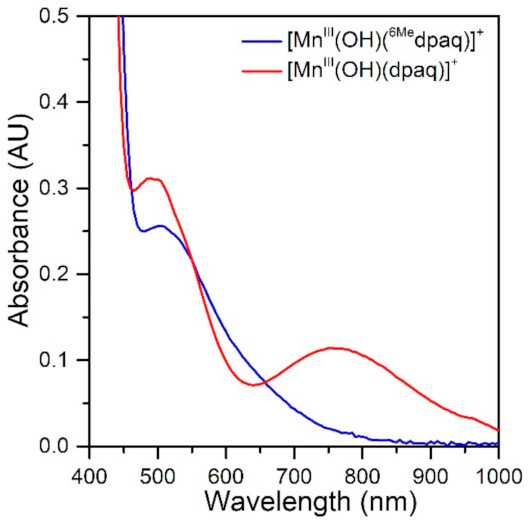
The electronic absorption spectrum of 1.0 mM [Mn^III^(OH)(^6Me^dpaq)]^+^ (blue trace) and 1.0 mM [Mn^III^(OH)(dpaq)]^+^ in MeCN at 25 °C shown for comparison (red trace).

The electronic absorption spectrum of [Mn^III^(OH)(^6Me^dpaq)]^+^ in MeCN at 25 °C shows a single broad absorption feature from 800 to 470 nm with *λ*_max_ at 510 nm (*ε* = 250 M^−1^ cm^−1^) (Figure 2). This spectrum deviates significantly from that observed for [Mn^III^(OH)(dpaq)]^+^ and its derivatives. The electronic absorption spectra of those complexes showed two absorption maxima with *λ*_max_ values at 770 and 500 nm (Figure 2) [17,18,20,46]. To understand the origin of the spectral perturbations for [Mn^III^(OH)(^6Me^dpaq)]^+^, we predicted the electronic absorption spectrum of this complex using time-dependent density functional theory (TD-DFT) calculations. Although TD-DFT calculations have known drawbacks, this method has performed exceptionally well for mononuclear Mn^III^ complexes [47,48], potentially because ligand-field transitions dominate the electronic absorption spectra of these complexes.

DFT geometry optimization for [Mn^III^(OH)(^6Me^dpaq)]^+^ and [Mn^III^(OH)(dpaq)]^+^ reproduced the trends in metric parameters obtained from X-ray diffraction (XRD) experiments for the complexes (Table 1). The Mn^III^-hydroxo distances (Mn−O1) for these complexes are essentially identical, while the greatest difference is in the Mn−N_pyridine_ distances (Mn−N4 and Mn−N5), which are elongated by 0.08 to 0.2 Å in [Mn^III^(OH)(^6Me^dpaq)]^+^. DFT calculations predict a (*d_xy_*)^1^(*d_yz_*)^1^(*d_xz_*)^1^(*d_x_*^2^*_-y_*^2^)^1^(*d_z_*^2^)^0^ ground configuration for each complex. (In the DFT computations for [Mn^III^(OH)(^6Me^dpaq)]^+^ and [Mn^III^(OH)(dpaq)]^+^, we chose a coordinate system whereby the *z*-axis lies along the Mn−O(H) bond and the *x*- and *y*-axes coincide with the equatorial Mn-ligand bonds. Accordingly, the *d_z_*^2^ and *d_x_*^2^*_−y_*^2^ MO are the σ-antibonding MOs, and the *d_xy_*, *d_yz_*, and *d_xz_* MOs are capable of π-interactions.) With this ground configuration, each spin-allowed ligand-field transition can be reasonably approximated by a one-electron excitation from a singly occupied Mn^III^ *d*-based MO to the unoccupied *d_z_*^2^-based MO. Consequently, the ligand-field electronic transition energies can be directly related to the Mn^III^ *d*-orbital splitting pattern. We will therefore briefly discuss the compositions and energies of the *d*-based MOs of [Mn^III^(OH)(^6Me^dpaq)]^+^ and [Mn^III^(OH)(dpaq)]^+^, as differences in these orbitals can account for all perturbations in the electronic absorption spectra of these complexes.

**Figure 3:**
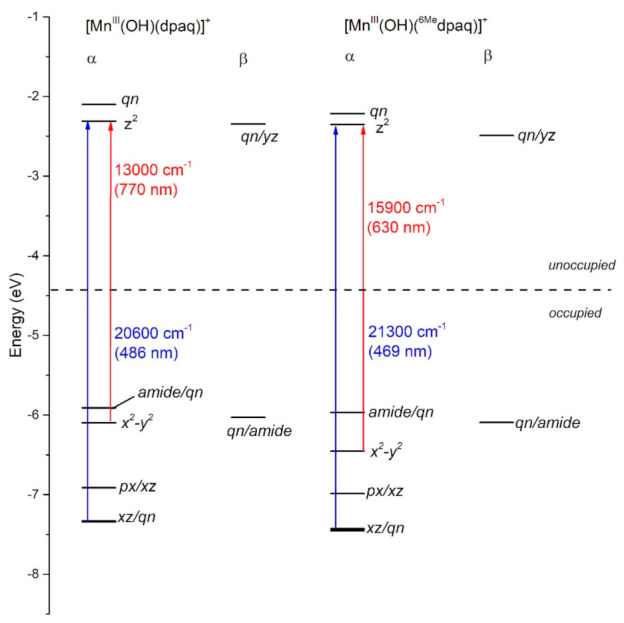
MO energy level diagram for [Mn^III^(OH)(dpaq)]^+^ and [Mn^III^(OH)(^6Me^dpaq)]^+^ based on the Kohn-Sham orbitals from DFT calculations. α and β refer to spin-up and spin-down MOs, respectively.

In each complex, the highest-energy *d_z_*^2^ MO is strongly destabilized by σ-antibonding interactions with both the hydroxo and carboxamido donors (Figure 3). The *d_z_*^2^ MO in each complex receives ligand contributions from the 2*p_x_*_,*y*_ orbitals of the equatorial N donor atoms, 2*p_z_* orbital of the carboxamido nitrogen atom and 2*p_z_* orbital of the oxygen atom of the hydroxo ligand (Table S1). The DFT calculations predict nearly identical energies (both roughly −2.2 eV; see Figure 3) for the *d_z_*^2^ MOs of [Mn^III^(OH)(^6Me^dpaq)]^+^ and [Mn^III^(OH)(dpaq)]^+^. Thus, any differences in the spectroscopic or chemical properties of these complexes are not found to be related to differences in Mn^III^-hydroxo or Mn^III^-amide bonding interactions. This DFT-based prediction is in accordance with the nearly identical Mn^III^−O_hydroxo_ (Mn−O1) and Mn^III^−N_amide_ (Mn−N2) bond lengths observed in both the experimental and DFT-computed structures of these complexes (Table 1). The energy and composition of the *d_x_*^2^_−*y*_^2^ MOs of [Mn^III^(OH)(^6Me^dpaq)]^+^ and [Mn^III^(OH)(dpaq)]^+^ show significantly higher levels of variation (Figure 3). The *d_x_*^2^_−*y*_^2^ MO is σ-antibonding with respect to the equatorial ligands and is therefore sensitive to the longer Mn^III^−N_pyridine_ bond lengths in [Mn^III^(OH)(^6Me^dpaq)]^+^ (Mn−N4 and Mn−N5; see Table 1). These longer, and therefore weaker Mn^III^−N_pyridine_ bonds lead to a stabilization of the *d_x_*^2^_−*y*_^2^ MO in [Mn^III^(OH)(^6Me^dpaq)]^+^ by 0.3 eV relative to that of [Mn^III^(OH)(dpaq)]^+^ (Figure 3). This stabilization creates a larger gap between the *d_z_*^2^ and *d_x_*^2^_−*y*_^2^ MOs of [Mn^III^(OH)(^6Me^dpaq)]^+^. The *d_xz_* and *d_yz_* MOs of each complex have weak π-antibonding interactions with the hydroxo ligand. The nearly identical Mn-hydroxo distances for the [Mn^III^(OH)(^6Me^dpaq)]^+^ and [Mn^III^(OH)(dpaq)]^+^ give rise to similar Mn-hydroxo π-interactions, causing the *d_xz_* and *d_yz_* MOs of these complexes to lie at similar energies. The *d_xy_* MO of each complex is non-bonding and, for this reason, the energy of this MO shows little variation between these complexes.

**Figure 4:**
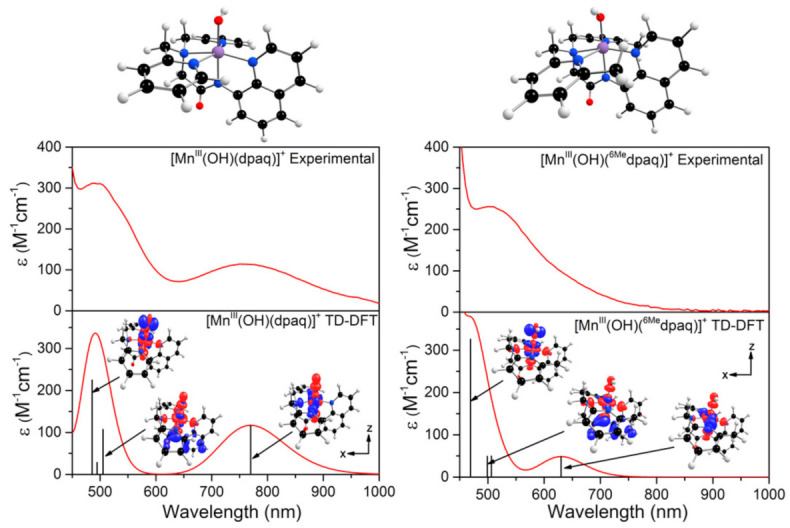
TD-DFT-computed electronic absorption spectra for [Mn^III^(OH)(dpaq)]^+^ (**left**) and [Mn^III^(OH)(^6Me^dpaq)]^+^ (**right**). The sticks indicate electronic transitions; EDDMs of selected transitions are included as an inset. Red and blue colors in the EDDMs denote gain and loss of electron density, respectively. The DFT-computed structures of the Mn^III^-hydoxo complexes are shown above the absorption spectra.

The TD-DFT-computed electronic absorption spectra of both [Mn^III^(OH)(^6Me^dpaq)]^+^ and [Mn^III^(OH)(dpaq)]^+^ are in excellent agreement with their experimental counterparts (Figure 4). Starting with [Mn^III^(OH)(dpaq)]^+^, the calculated spectrum shows two bands at 770 and 500 nm, which nearly perfectly reproduce the experimental spectrum (Figure 4, left). An analysis of the electron-density difference maps (EDDMs), illustrating the factors contributing to these bands, shows that each band derives from Mn^III^ ligand-field transitions. The lower-energy band at 770 nm is a result of a one-electron *d_x_*^2^_−*y*_^2^ → *d_z_*^2^ transition. The band at 500 nm results from two ligand-field transitions—a *d_yz_* → *d_z_*^2^ transition at 505 nm and a *d_xz_* → *d_z_*^2^ transition near 486 nm. Given these assignments, the position of the lower-energy band reflects the difference between Mn-ligand *σ*-interactions with the axial and equatorial ligands, while the energy of the higher-energy band elucidates the differences between Mn-hydroxo *σ*- and *π*-interactions.

The TD-DFT-computed electronic absorption spectrum of [Mn^III^(OH)(^6Me^dpaq)]^+^ also reproduces the experimental spectrum well (Figure 4). The computed spectrum shows a weak feature near 630 nm that corresponds with a distinct rise of the absorption intensity in the experimental spectrum. The calculated spectrum also shows a greater intensity at wavelengths less than 550 nm, in good agreement with experiment (Figure 4, right). Importantly, the TD-DFT-computed spectrum of [Mn^III^(OH)(^6Me^dpaq)]^+^ reproduces all the major differences and similarities relative to [Mn^III^(OH)(dpaq)]^+^; i.e., the lowest-energy band has a pronounced blue-shift, while the higher-energy bands are relatively unperturbed. The lowest-energy band of [Mn^III^(OH)(^6Me^dpaq)]^+^ at 630 nm indicates a one-electron *d_x_*^2^_−*y*_^2^ → *d_z_*^2^ transition. The pronounced blue-shift relative to that of [Mn^III^(OH)(dpaq)]^+^ (770 to 630 nm) can be rationalized on the basis of the stabilization of the *d_x_*^2^_−*y*_^2^ MO of [Mn^III^(OH)(^6Me^dpaq)]^+^, caused by the longer Mn^III^−N_pyridine_ bond lengths (Figure 3 and Table 1). The higher-energy band in the TD-DFT-computed spectrum of [Mn^III^(OH)(^6Me^dpaq)]^+^ contains the *d_yz_* → *d_z_*^2^ and *d_xz_* → *d_z_*^2^ transitions. The wavelengths of these transitions are only slightly blue-shifted relative to the corresponding transitions in [Mn^III^(OH)(dpaq)]^+^ (505 vs. 499 nm, and 486 vs. 469 nm, respectively; see Figure 4), which is in agreement with the minor perturbations in the energies of these MOs (Figure 3). Overall, the TD-DFT computations reveal that the spectral perturbations between [Mn^III^(OH)(dpaq)]^+^ and [Mn^III^(OH)(^6Me^dpaq)]^+^ can be understood on the basis of the elongated Mn^III^−N_pyridine_ bonds in the latter complex.

### 3.3. Oxidative Reactivity of [Mn^III^(OH)(^6Me^dpaq)](OTf)

**Figure 5:**
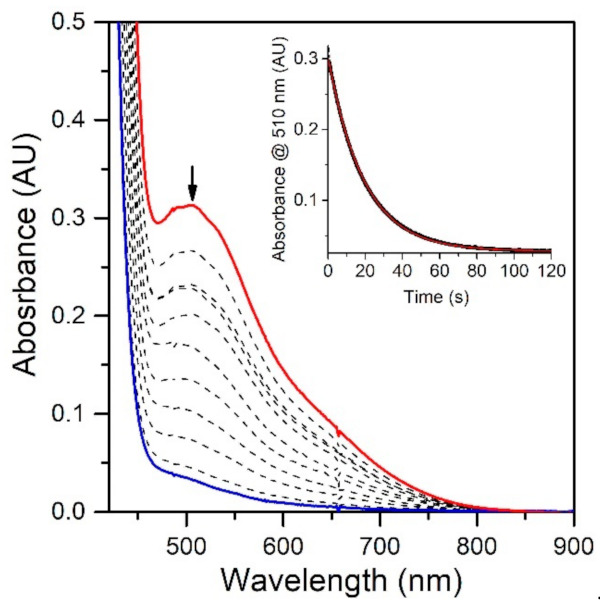
Reactions of 1.25 mM [Mn^III^(OH)(^6Me^dpaq)](OTf) with 10 equiv. TEMPOH at −35 °C in MeCN (initial and final spectra are the red and blue traces, respectively). Inset: The decay of the 510 nm band over time (black trace) and fit to pseudo-first-order kinetic model (red trace).

**Figure 6:**
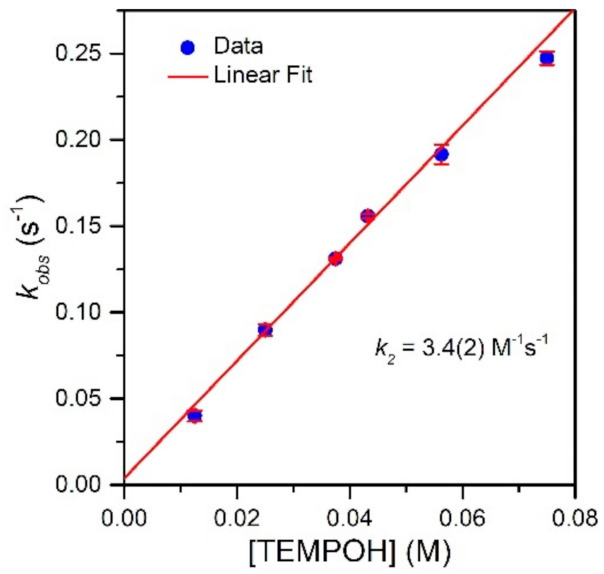
Pseudo-first-order rate constants *k_obs_* (s^−1^) as a function of TEMPOH concentration for a 1.25 mM solution of [Mn^III^(OH)(^6Me^dpaq)](OTf) in MeCN at −35 °C. The second-order rate constant (*k*_2_) was calculated from the slope of the linear fit.

To evaluate the effect of the Mn^III^−N_pyridine_ bond elongations of [Mn^III^(OH)(^6Me^dpaq)]^+^ on chemical reactivity, we explored the reactions of this complex with TEMPOH and xanthene. The addition of 10 equiv. TEMPOH to a 1.25 mM solution of [Mn^III^(OH)(^6Me^dpaq)]^+^ in MeCN at −35 °C led to the disappearance of the electronic absorption features of the Mn^III^-hydroxo complex within 100 s, providing a final spectrum identical to that of [Mn^II^(H_2_O)(^6Me^dpaq)]^+^ with approximately 100% yield (Figure 5). An EPR analysis of the solution, conducted following the reaction, reveals an intense signal from TEMPO radical (Figure S5). The observed products are consistent with a CPET reaction between the Mn^III^-hydroxo center and TEMPOH, which would afford TEMPO radical and [Mn^II^(H_2_O)(^6Me^dpaq)]^+^. Kinetic experiments were performed at −35 °C using 10–60 equiv. TEMPOH to obtain the second-order rate constant (*k*_2_). A plot of *k_obs_* vs. the concentration of TEMPOH is linear, and a fit to these data provided the second-order rate constant (*k*_2_) of 3.4(2) M^−1^ s^−1^ at −35 °C (Figure 6). 

Table 2 compares the second-order rate constant for TEMPOH oxidation by [Mn^III^(OH)(^6Me^dpaq)]^+^ with the rate constant determined for [Mn^III^(OH)(dpaq)]^+^ and its derivatives. The rate constant of TEMPOH oxidation for [Mn^III^(OH)(^6Me^dpaq)]^+^ is nearly three-fold faster than that of [Mn^III^(OH)(dpaq)]^+^ (*k*_2_ = 1.1(1) M^−1^ s^−1^) and almost the same as that determined for [Mn^III^(OH)(dpaq^2Me^)]^+^ (*k*_2_ = 3.9(3) M^−1 ^s^−1^) [17,20]. Thus, Me substituents on the pyridyl or quinolinyl groups have similar effects on reactivity.

We also investigated the reactivity of [Mn^III^(OH)(^6Me^dpaq)]^+^ with xanthene by treating a 1.25 mM solution of this Mn^III^-hydroxo complex in MeCN with 250 equiv. xanthene at 50 °C. In this case, the absorbance band of [Mn^III^(OH)(^6Me^dpaq)]^+^ at 510 nm decayed by only 10% over the course of 1000 min, which is within the range of the self-decay rate for [Mn^III^(OH)(^6Me^dpaq)]^+^ at this temperature. The apparent lack of reactivity is somewhat unexpected, as [Mn^III^(OH)(dpaq)]^+^ reacts with xanthene under similar conditions, albeit at a slow rate (0.0008 s^−1^). It is possible that the decreased reactivity for [Mn^III^(OH)(^6Me^dpaq)]^+^ might be due to the steric bulk of the 6-methyl-pyridyl substituents, which could hinder the access of xanthene to the Mn^III^-hydroxo unit. Notably, [Mn^III^(OH)(dpaq^2Me^)]^+^ reacts with xanthene three times slower than [Mn^III^(OH)(dpaq)]^+^ (0.00025 s^−1^ vs. 0.0008 s^−1^, respectively).^17^ DFT computations demonstrated that the steric bulk of the 2-Me-quinoline moiety in [Mn^III^(OH)(dpaq^2Me^)]^+^ caused xanthene to orientate differently in the transition state when compared to [Mn^III^(OH)(dpaq)]^+^. This reorientation introduced a destabilizing effect that led to a higher transition state of [Mn^III^(OH)(dpaq^2Me^)]^+^ than that of [Mn^III^(OH)(dpaq)]^+^ by around 3 kcal/mol. A similar situation, caused by the 6-Me-pyridyl groups of [Mn^III^(OH)(^6Me^dpaq)]^+^, could hamper the reaction of this complex with xanthene.

### 3.4. Thermodynamic Driving Force for TEMPOH Oxidation Using Experimental and Computational Methods

CPET reaction rates of Mn^III^-hydroxo complexes show a strong correlation to the thermodynamic driving force [20]. For these reactions, the driving force is the difference between the BDFE of the Mn^II^O(H)-H bond that is formed, and the TEMPO-H bond that is broken (Equation (1)).

Δ*G* = BDFE(Mn^II^O(H)-H)-BDFE(TEMPO-H) (1)

**Scheme 3:**
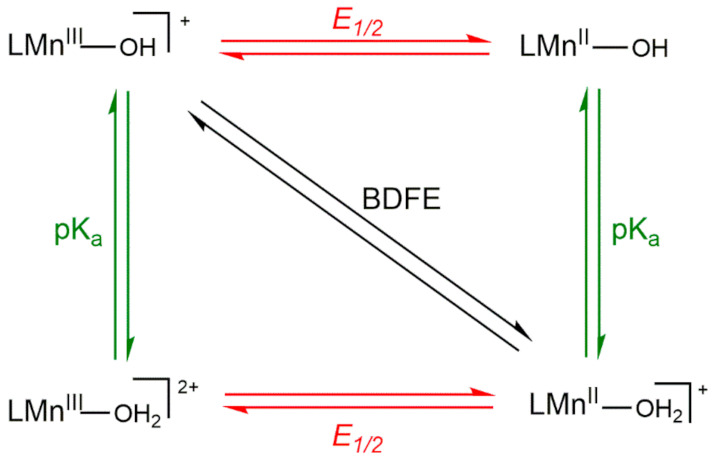
Thermodynamic Square Scheme for Decomposing the O−H BDFE of a Mn^III^-OH/Mn^II^-OH_2_ Couple.

Thus, the variation in driving force among a set of Mn^III^-hydroxo complexes arises from the O−H BDFE of the Mn^II^-aqua complex. To understand the reason behind changes in the O−H BDFE, it is helpful to deconstruct the BDFE into individual proton- and electron-transfer steps, as represented by the square diagram (Scheme 3). The reactions along the top and bottom edges represent single electron-transfer steps, and reactions along the left and right edges represent single proton-transfer steps. The diagonal line represents CPET. The O−H BDFE of a Mn^II^-aqua complex can be calculated using the one-electron reduction potential of the Mn^III/II^-OH couple and the p*K*_a_ of the Mn^II^-OH_2_ product using the modified Bordwell equation (Equation (2)) [49].

BDFE(Mn^II^O(H)-H) = 1.37p*K*_a_ + 23.06*E*_1/2_ + C_G,sol_ (2)

In this equation, C_G,sol_ is a constant for a given solvent (C_G,sol, MeCN_ = 54.9 kcal/mol). In our previous investigation of [Mn^III^(OH)(dpaq^5R^)]^+^ complexes, we observed that both the reduction potential and p*K*_a_ changed as a function of the R substituent, although the potential changed more dramatically and was, therefore, largely responsible for the net change in O−H BDFE [20].

We performed cyclic voltammetry (CV) experiments of [Mn^III^(OH)(^6Me^dpaq)]^+^ to understand the thermodynamic effects on the CPET reactivity of the complex . When scanning to negative potentials, we observed a reduction wave (*E*_p,c_) at −0.63 V Fc^+^/Fc (Figure S6) that we attributed to a reduction of the Mn^III^-hydroxo complex. This potential is between those of [Mn^III^(OH)(dpaq^2Me^)]^+^ and [Mn^III^(OH)(dpaq^5Cl^)]^+^ (−0.62 V and −0.66 V vs. Fc^+^/Fc, respectively) [20]. The *E*_p,c_ value for [Mn^III^(OH)(^6Me^dpaq)]^+^ and those previously measured for [Mn^III^(OH)(dpaq)]^+^ and its derivatives are presented in Table 2.

Following a previous approach [20], we also calculated the p*K_a_* and *E*_1/2_ values associated with the [Mn^III^(OH)(^6Me^dpaq)]^+^/[Mn^II^(OH_2_)(^6Me^dpaq)]^+^ couple. The details of these calculations are discussed in the Supplementary Materials and the calculated values are presented in Table 2. The *E*_1/2_ value of −0.60 V for the [Mn^III^(OH)(^6Me^dpaq)]^+^/[Mn^II^(OH_2_)(^6Me^dpaq)]^+^ couple is 30 mV higher than the experimental peak potential. This calculated *E*_1/2_ value is 100 mV greater than that of [Mn^III^(OH)(dpaq)]^+^ (−0.70 V) but nearly the same as that of [Mn^III^(OH)(dpaq^2Me^)]^+^ (−0.58 V). Thus, [Mn^III^(OH)(^6Me^dpaq)]^+^ is found to be a slightly better one-electron oxidant than [Mn^III^(OH)(dpaq)]^+^ and is equal to [Mn^III^(OH)(dpaq^2Me^)]^+^. The increased Mn^III/II^ reduction potential in [Mn^III^(OH)(^6Me^dpaq)]^+^, relative to [Mn^III^(OH)(dpaq)]^+^, results from the longer Mn^III^−N_pyridine_ bonds (Mn−N4 and Mn−N5, see Table 1). This elongation reduces the interaction between the Mn center and the pyridine ligands, which consequentially leads to a more Lewis acidic Mn center with a higher propensity for one-electron reduction. The DFT calculations predict a p*K*_a_ value of 28.6 for [Mn^II^(OH_2_)(^6Me^dpaq)]^+^, which makes this complex slightly more acidic than [Mn^II^(OH_2_)(dpaq)]^+^ (p*K*_a_ = 29.3). Together the *E*_1/2_ and p*K*_a_ values combine to yield a O−H BDFE of 80.2 kcal/mol for [Mn^II^(OH_2_)(^6Me^dpaq)]^+^, which is larger than that of [Mn^III^(OH)(dpaq)]^+^ (79.1 kcal/mol). It is important to note that the *E*_1/2_ and p*K*_a_ terms for [Mn^II^(OH_2_)(^6Me^dpaq)]^+^ shift in directions that oppose one another when considering the net BDFE, but the shift in *E*_1/2_ is larger and therefore dominant. More specifically, while [Mn^II^(OH_2_)(^6Me^dpaq)]^+^ is less basic than [Mn^III^(OH)(dpaq)]^+^ by 0.7 p*K*_a_ units (0.96 kcal/mol contribution to BDFE), the Mn^III^ center in [Mn^III^(OH)(^6Me^dpaq)]^+^ has a more positive *E*_1/2_ than [Mn^III^(OH)(dpaq)]^+^ by 0.10 V (2.08 kcal/mol contribution to BDFE).

**Figure 7:**
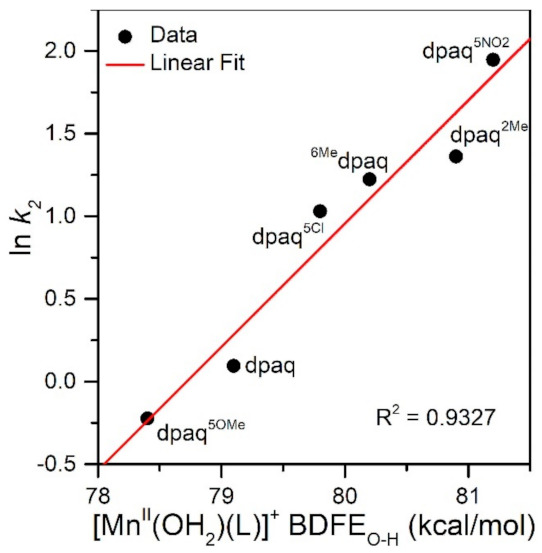
A plot of ln *k*_2_ vs. Mn^II^-aqua O−H BDFE (kcal/mol).

**Figure 8:**
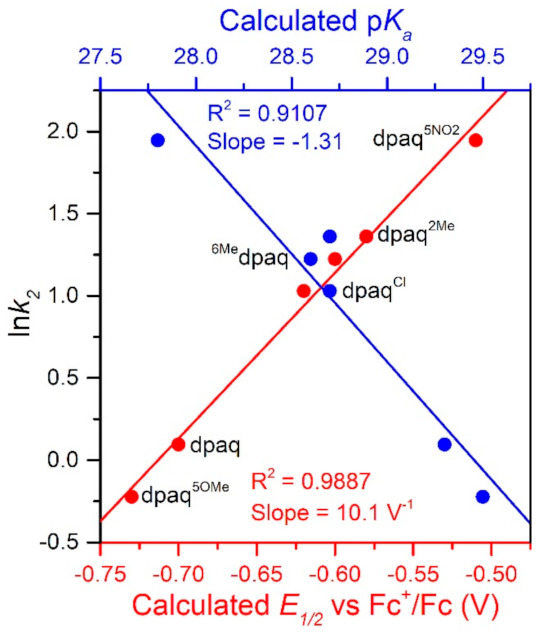
A plot of ln *k*_2_ vs. calculate *E*_1/2_ vs. Fc^+^/Fc (V) and p*K_a_*.

The rate of TEMPOH oxidation by [Mn^III^(OH)(^6Me^dpaq)]^+^ can be plotted against the O−H BDFE of the [Mn^II^(OH_2_)(^6Me^dpaq)]^+^ product (Figure 7). As shown in this plot, the [Mn^III^(OH)(^6Me^dpaq)]^+^ complex follows the linear correlation previously observed for Mn^III^-hydroxo complexes supported by the dpaq ligand and its derivatives quite well [20]. The individual contributions of the p*K_a_* and *E*_1/2_ to the Mn^II^-aqua O−H BDFEs for these complexes are presented in Figure S7, and the correlations of these parameters with the reaction rates of the Mn^III^-hydroxo complexes with TEMPOH are shown in Figure 8. Among this series, the more significant change to BDFE originates from the *E*_1/2_ factors, with only a slight effect enacted by the p*K_a_* (Table 2 and Figure 8). The p*K_a_* change across the series is 1.7 p*K_a_* units (2.33 kcal/mol), whereas the calculated *E*_1/2_ changes by 0.22 V (5.07 kcal/mol). Thus, the *E*_1/2_ contributes around twice as much as the p*K_a_* to the net BDFE across the series.

From Figure 8, we observe a strong linear correlation between the *E*_1/2_ of the Mn^III/II^ couple and the ln(*k*_2_) for TEMPOH oxidation, with more positive potentials translating to faster reaction rates. In contrast, the plot of ln(*k*_2_) for TEMPOH oxidation versus the Mn^II^-aqua p*K*_a_ value shows an inverse correlation, where the more basic complexes experience slower reaction rates. Thus, the p*K_a_* and *E*_1/2_ counterbalance each other to affect the rate of reaction of the O−H BDFE. 

### 3.5. Ligand Substitution Reactions of [Mn^III^(OO^t^Bu)(^6Me^dpaq)]^+^ with Water

**Figure 9:**
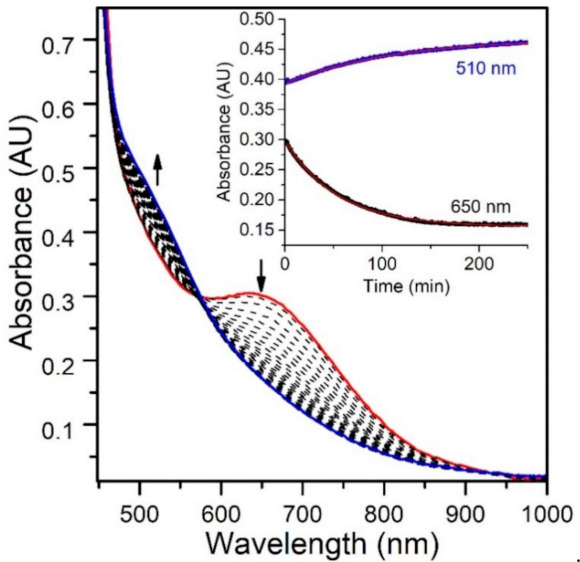
Electronic absorption spectra monitoring the reaction of an anaerobic sample of 2 mM [Mn^III^(OO*^t^*Bu)(^6Me^dpaq)]^+^ in MeCN with 100 equiv. of H_2_O at 298 K. (Inset) time course for the spectral changes.

**Figure 10:**
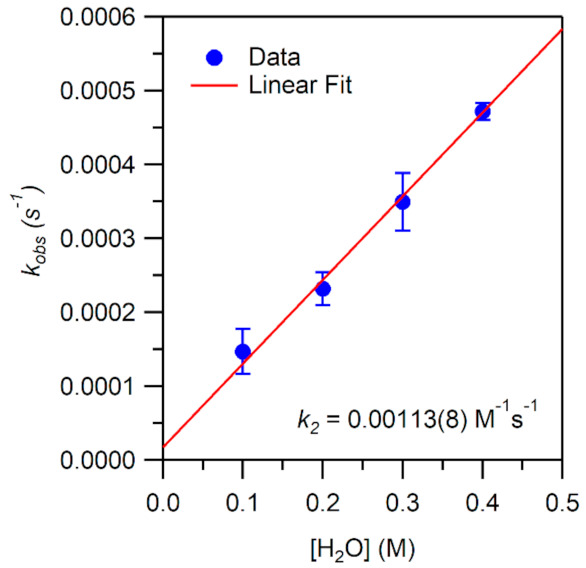
Pseudo-first-order rate constants *k_obs_* (s^−1^) as a function of H_2_O concentration for an anaerobic solution of 1 mM [Mn^III^(OO^tBu^)(^6Me^dpaq)]^+^ in MeCN at 25 °C. The second-order rate constant (*k*_2_) was calculated from the slope of the linear fit.

While the reaction of TEMPOH with [Mn^III^(OH)(^6Me^dpaq)]^+^ mimics one of the elementary reactions proposed in the catalytic cycle of MnLOX, one of the proposed mechanisms for this enzyme postulates a ligand substitution reaction, where a Mn^III^-alkylperoxo complex reacts with water to give the alkyl hydroperoxo product and a Mn^III^-hydroxo complex [50]. Since both [Mn^III^(OH)(^6Me^dpaq)]^+^ and its Mn^III^-alkylperoxo analogue [Mn^III^(OO*^t^*Bu)(^6Me^dpaq)]^+^ are well characterized and stable at room temperature, we took advantage of these complexes to explore whether we could mimic this proposed ligand substitution reaction. The addition of 100 equiv. H_2_O to [Mn^III^(OO*^t^*Bu)(^6Me^dpaq)]^+^ in an anerobic solution of MeCN resulted in a loss of intensity of the electronic absorption band of the Mn^III^-alkylperoxo complex at 650 nm and growth in absorbance at ~500 nm (Figure 9). This conversion demonstrates isosbestic behavior (with an isosbestic point at ~574 nm), suggesting a simple conversion that does not involve accumulating intermediates. The final spectrum is identical to that of [Mn^III^(OH)(^6Me^dpaq)]^+^, consistent with a ligand substitution reaction. The decay of the optical signal of [Mn^III^(OO*^t^*Bu)(^6Me^dpaq)]^+^ in the presence of H_2_O (650 nm) follows pseudo-first order behavior (Figure 9, inset), allowing us to determine a pseudo-first order rate constant (*k*_obs_). Experiments using different concentrations of H_2_O showed a linear increase in *k*_obs_ with increasing water concentrations. An analysis of these data yield a second order rate constant for the ligand substitution reaction of 1.13(8) × 10^−3^ M^−1^ s^−1^ at 25 °C (Figure 10). A mass spectral analysis of the solution following the reaction of [Mn^III^(OO*^t^*Bu)(^6Me^dpaq)]^+^ with H_2_O revealed a peak at *m/z* = 482.14 (Figure S8), further confirming the formation of [Mn^III^(OH)(^6Me^dpaq)]^+^ (calculated *m*/*z* = 482.14). At longer time periods, we observed the precipitation of a brown solid, which may provide evidence of a degree of demetallation of the [Mn^III^(OH)(^6Me^dpaq)]^+^ due to the presence of water.

## 4. Conclusions

In this study, we examined the spectroscopic properties and chemical reactivity of the Mn^III^-hydroxo complex [Mn^III^(OH)(^6Me^dpaq)]^+^. Using TD-DFT computations, we were able to explain differences in the electronic absorption spectra of [Mn^III^(OH)(^6Me^dpaq)]^+^ and [Mn^III^(OH)(dpaq)]^+^ in terms of the stabilization of the Mn *d_x_*^2^_-*y*_^2^ orbital in the former complex. Orbital stabilization occurs due to longer Mn^III^−N_pyridine_ bonds, caused by the 6-Me-pyridyl groups in [Mn^III^(OH)(^6Me^dpaq)]^+^. Importantly, DFT computations predict no difference in the energy of the redox-active *d_z_*^2^ MO between the [Mn^III^(OH)(^6Me^dpaq)]^+^ and [Mn^III^(OH)(dpaq)]^+^ complexes. Thus, any differences in redox reactivity between these complexes must stem from factors beyond the energy of the *d_z_*^2^ MO. The oxidative capability of the [Mn^III^(OH)(^6Me^dpaq)]^+^ complex was assessed by exploring the rate of TEMPOH oxidation. The reactivity of [Mn^III^(OH)(^6Me^dpaq)]^+^ with TEMPOH is 3-fold faster than that of [Mn^III^(OH)(dpaq)]^+^. This rate enhancement results from the greater Mn^III/II^ reduction potential of [Mn^III^(OH)(^6Me^dpaq)]^+^ than [Mn^III^(OH)(dpaq)]^+^. The TEMPOH oxidation rate for [Mn^III^(OH)(^6Me^dpaq)]^+^ is in accordance with a previously established linear free energy relationship for Mn^III^-hydroxo complexes [20]. The related [Mn^III^(OO*^t^*Bu)(^6Me^dpaq)]^+^ complex reacts with water to form the Mn^III^-hydroxo complex [Mn^III^(OH)(^6Me^dpaq)]^+^, providing a mimic of an elementary step proposed in some mechanisms for MnLOX.

## Figures and Tables

**Table 1:** Selected Manganese−Ligand Bond Lengths (Å) for [Mn^III^(OH)(dpaq)]^+^ and [Mn^III^(OH)(^6Me^dpaq)]^+^ from X-ray Crystallography and DFT Computations.

	[Mn^III^(OH)(dpaq)]^+^		[Mn^III^(OH)(^6Me^dpaq)]^+^	
	XRD *^a^*	DFT	XRD *^b^*	DFT
Mn−O1 (Å)	1.806(13)	1.829	1.806(6)	1.830
Mn−N1 (Å)	2.072(14)	2.089	2.041(7)	2.048
Mn−N2 (Å)	1.975(14)	1.980	1.962(6)	1.968
Mn−N3 (Å)	2.173(14)	2.222	2.130(6)	2.134
Mn−N4 (Å)	2.260(14)	2.213	2.322(6)	2.339
Mn−N5 (Å)	2.216(15)	2.209	2.381(7)	2.422

*^a^* From [18]. *^b^* From [30].

**Table 2:** Second-Order Rate Constants (*k*_2_) for TEMPOH Oxidation by Mn^III^-hydroxo Complexes, Experimental *E_p,c_* Values, and DFT-Calculated Thermodynamic Parameters.

	Experimental	DFT-Calculated			
Complex	k_2_ (M^−1^s^−1^)	Mn^III^/^II^ E_p,c_ ^a^	Mn^III^/^II^ E_1/2_ ^a^	Mn^II^-OH_2_ pK_a_	BDFE ^b^
[Mn^III^(OH)(^6Me^dpaq)]^+^	3.4(2)	−0.63	−0.60	28.6	80.2
[Mn^III^(OH)(dpaq)]^+ c^	1.1(1)	−0.70	−0.70	29.3	79.1
[Mn^III^(OH)(dpaq^2Me^)]^+ c^	3.9(3) ^d^	−0.62 ^d^	−0.58 ^c^	28.7 ^c^	80.9 ^c^
[Mn^III^(OH)(dpaq^5NO2^)]^+ c^	7(1)	−0.57	−0.51	27.8	81.2
[Mn^III^(OH)(dpaq^5Cl^)]^+ c^	2.8(2)	−0.66	−0.62	28.7	79.8
[Mn^III^(OH)(dpaq^5OMe^)]^+ c^	0.8(1)	−0.72	−0.73	29.5	78.4

^a^ In V relative to Fc^+^/Fc. ^b^ In kcal mol^−1^. ^c^ From [20]. ^d^ From [17].

## Data Availability

Raw data are available upon request.

## Supplementary Materials

The following are available online, Figure S1: Electronic absorption spectra showing the reaction of [Mn^III^(OO*^t^*Bu)(^6Me^dpaq)]^+^ in TFE at 50 °C to form the [Mn^III^(OCH_2_CF_3_)(^6Me^dpaq)]^+^ complex (blue tace). Figure S2: ESI-MS data for [Mn^III^(OO*^t^*Bu)(^6Me^dpaq)]^+^ in TFE. Figure S3: Left: Electronic absorption spectra of the dissolution of [Mn^III^(OO*^t^*Bu)(^6Me^dpaq)]^+^ in MeOH. Right: Reaction of [Mn^III^(OO*^t^*Bu)(^6Me^dpaq)]^+^ in MeCN with MeOH under anaerobic conditions. Figure S4: ESI-MS data for [Mn^III^(OO*^t^*Bu)(^6Me^dpaq)](OTf) in MeOH. Figure S5: EPR spectrum of the solution following reaction of [Mn^III^(OH)(^6Me^dpaq)]^+^ and TEMPOH. Figure S6: Cyclic voltammetry trace of [Mn^III^(OH)(^6Me^dpaq)]^+^. Figure S7: Thermodynamic contributions to the O−H BDFE of Mn^II^-aqua complexes from the Mn^III^-OH/Mn^II^-OH reduction potentials and Mn^II^-aqua p*K*_a_ values. Figure S8: ESI-MS data for [Mn^III^(OO*^t^*Bu)(^6Me^dpaq)]^+^ with H_2_O. Table S1: TD-DFT calculated energies, percent contributions from dominant one-electron excitations, and oscillator strengths for the major electronic transitions in [Mn^III^(OH)(dpaq)]^+^ and [Mn^III^(OH)(^6Me^dpaq)]^+^. Table S2: Calculated energies (eV) and contributions (%) of Mn 3*d*, O and N 2*p*-based MOs of [Mn^III^(OH)(dpaq)]^+^ and [Mn^III^(OH)(^6Me^dpaq)]^+^. Table S3: DFT-Calculated Δ*G* values (in kcal/mol) for the addition of a proton or electron, used in calculating the *E*_red_ and p*K*_a_ values. Table S4: DFT-calculated thermodynamic parameters used to determine Mn^II^-OH_2_ BDFEs for the [Mn^II^(OH_2_)(^6Me^dpaq)]^+^ complex. Tables S5–S9: Cartesian coordinates for DFT-optimized structures.

## Sample Availability

Samples of the Mn coordination compounds are available from the authors.
