# Supplementary material for: Mimicking Elementary Reactions of Manganese Lipoxygenase Using Mn-hydroxo and Mn-alkylperoxo Complexes
Source: Molecules. 2021 Nov 25;26(23):7151. doi: 10.3390/molecules26237151 (PMC8659247; doi:10.3390/molecules26237151)
Supplement: Supplementary file 1 [file molecules-26-07151-s001.zip › molecules-1461647-supplementary.pdf]

Supplementary Materials for

Mimicking Elementary Reactions of Manganese Lipoxygenase using Mn-hydroxo and Mn-alkylperoxo Complexes

Adedamola A. Opalade,[a] Elizabeth N. Grotemeyer,[a] and Timothy A. Jackson*[a]

[a] *The University of Kansas, Department of Chemistry and Center for Environmentally Beneficial Catalysis, 1567 Irving Hill Road, Lawrence, KS 66045, USA.*

*To whom correspondence should be addressed:
Timothy A. Jackson
Phone: (785) 864-3968
taj@ku.edu

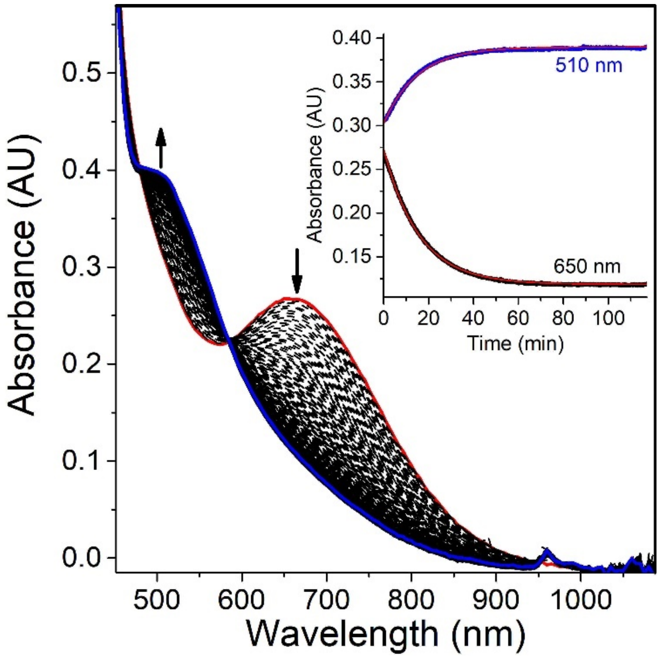

Figure S1. Electronic absorption spectra monitoring the reaction of anaerobic sample of 1.75 mM $[Mn^{III}(OO^tBu)(^{6Me}dpaq)]^+$ in TFE (red trace) at 50 °C to form the $[Mn^{III}(OCH_2CF_3)(^{6Me}dpaq)]^+$ complex (blue tace). The inset shows the timecourse for spectral changes at 510 and 650 nm (blue and black dots, respectively) and fits to a first-order model (red traces).

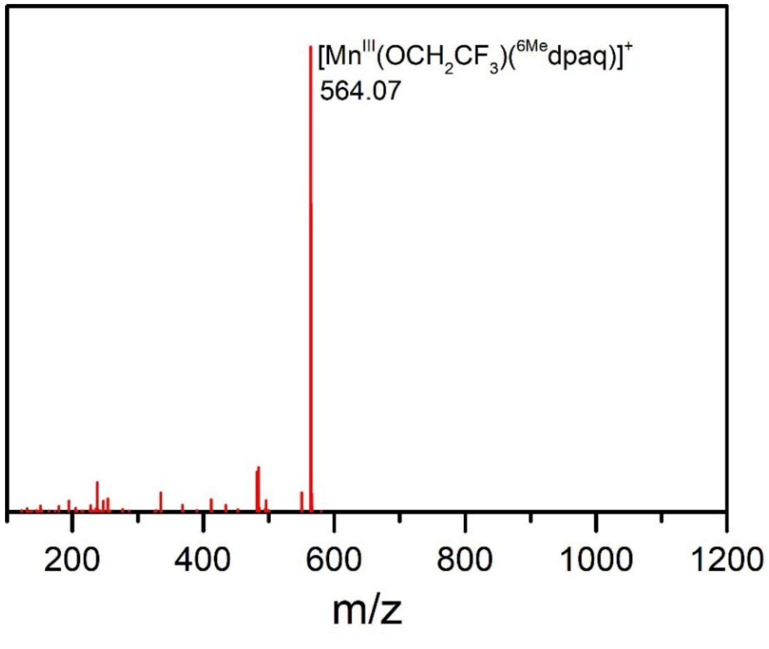

Figure S2. ESI-MS data for a 2×10^{-3} mM solution of $[Mn^{III}(OO^tBu)(^{6Me}dpaq)]^+$ in TFE prepared under anaerobic conditions.

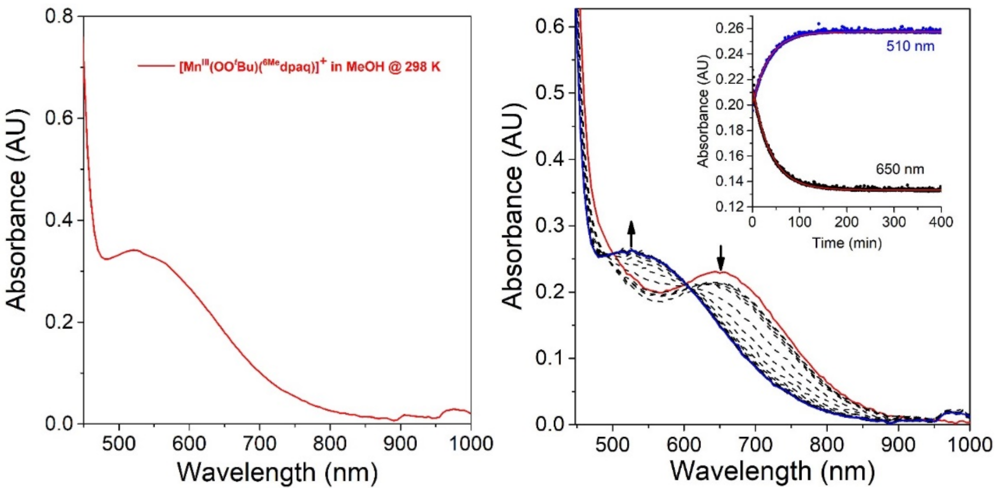

Figure S3. Left: Electronic absorption spectra of final reaction mixture from the dissolution of [Mn^{III}(OO^{t}Bu)(^{6Me}dpaq)]$^+$ in MeOH under anaerobic conditions at 25 °C. Right: Reaction of 1.0 mM solution of [Mn^{III}(OO^{t}Bu)(^{6Me}dpaq)]$^+$ in MeCN with 100 μL MeOH under anaerobic conditions at 25 °C. The inset shows the timecourse for spectral changes at 510 and 650 nm (blue and black dots, respectively) and fits to a first-order model (red traces).

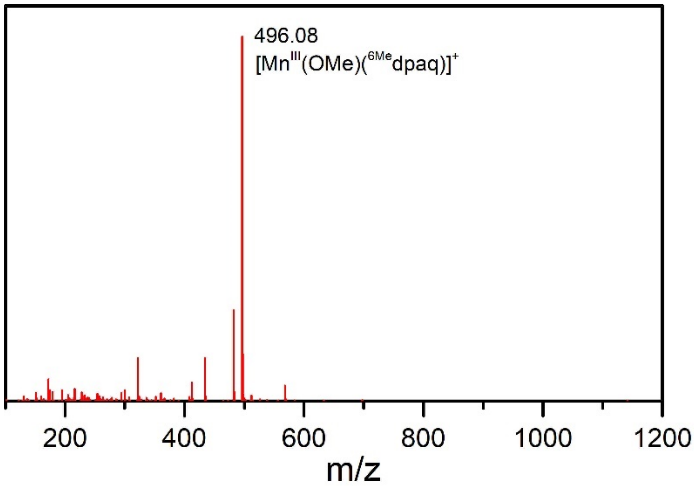

Figure S4. ESI-MS data for a 2 × 10^{-3} mM solution of [Mn^{III}(OO^{t}Bu)(^{6Me}dpaq)](OTf) prepared in MeOH under anaerobic conditions.

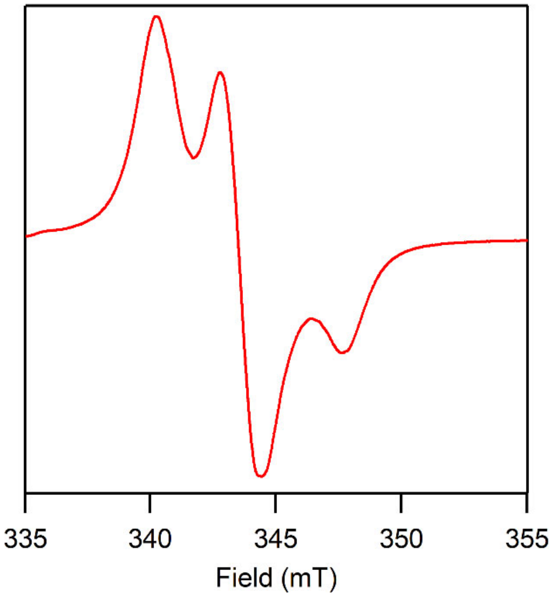

Figure S5. Perpendicular-mode EPR spectrum (8 K) of an MeCN solution following the reaction of 1.25 mM $[Mn^{III}(OH)(^{6Me}dpaq)]^+$ with 100 equiv. TEMPOH. The intense signal from 340 - 350 mT is attributed to the TEMPO radical.

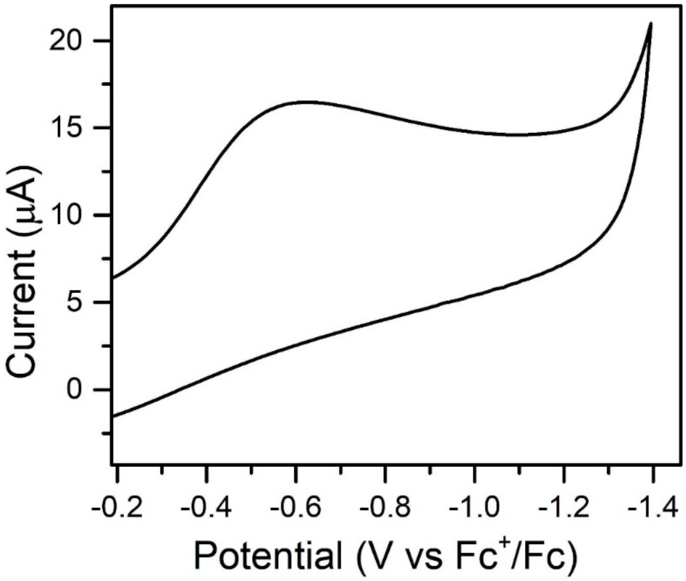

Figure S6. Cyclic voltammetry trace of $[Mn^{III}(OH)(^{6Me}dpaq)]^+$ showing the Mn^{III}/Mn^{II} wave with a scan rate of 100 mV s^{-1}. The working electrode was a glassy carbon electrode with a Pt wire as the counter electrode. Ag/AgCl quasi-reference electrode was used, and Fc^+/Fc potential was measured as an external reference. 2 mM solutions of $[Mn^{III}(OH)(^{6Me}dpaq)]^+$ prepared from 0.1 M Bu_4NPF_6 electrolyte solution in CH_3CN were used for measurements at 25 °C.

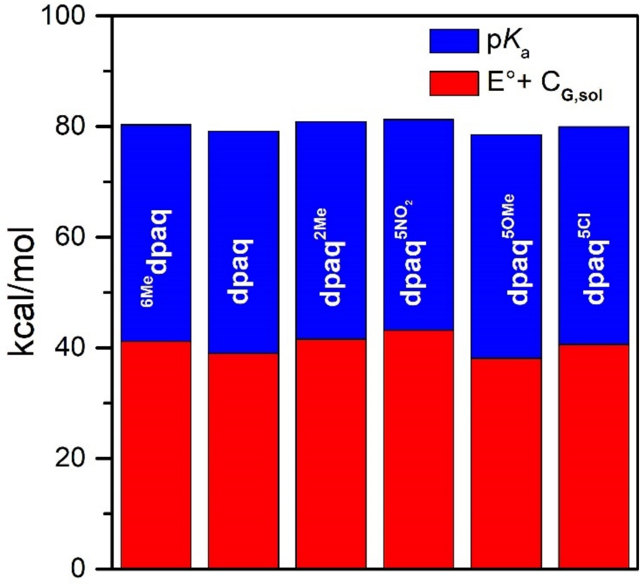

Figure S7. Thermodynamic contributions to the O–H BDFE of Mn^{II}-aqua complexes from the Mn^{III}-OH/Mn^{II}-OH reduction potentials and Mn^{II}-aqua pK_a values.

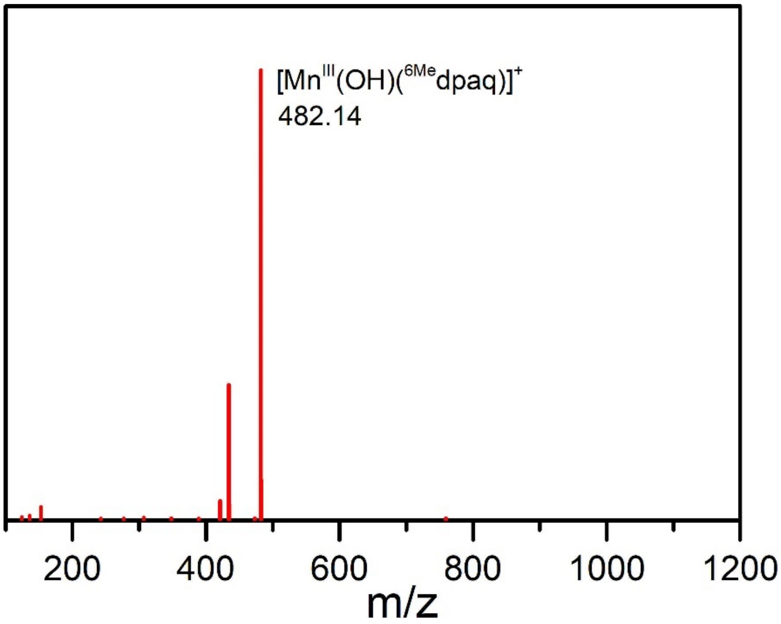

Figure S8. ESI-MS data for a 2×10^{-3} mM solution after the reaction of $[Mn^{III}(OO^tBu)(^{6Me}dpaq)]^+$ with H_2O under anaerobic conditions at 25 °C.

Table S1. Calculated energies (eV) and contributions (%) of Mn 3*d*, O and N 2*p*-based spin-up MOs of $[Mn^{III}(OH)(dpaq)]^{+}$ and $[Mn^{III}(OH)(^{6Me}dpaq)]^{+}$.

	MO	Spin	Occ.	Energy (eV)	% Contribution		
					Mn 3*d*	N 2*p*	OH
dpaq	114	α	1	-7.3415	13.2	6.90	41
	115	α	1	-7.3322	4.5	2.20	11.8
	116	α	1	-6.9145	8.3	9.9	11.0
	117	α	1	-6.0988	40.4	36.9	0.6
	118	α	1	-5.9111	2.5	23.5	0.1
	119	α	0	-2.3101	57.3	16.7	11.6
^{6Me}dpaq	121	α	1	-7.4285	14.3	3.3	42.7
	122	α	1	-7.2994	1.2	1.4	0.6
	123	α	1	-7.1537	0.0	0.0	0.0
	124	α	1	-6.9890	5.6	6.3	10.4
	125	α	1	-6.4543	37.0	14.7	2.0
	126	α	1	-5.9682	2.9	23.5	0.2
	127	α	0	-2.3528	54.3	12.0	9.6
	128	α	0	-2.2140	2.8	16.6	0.4

Table S2. TD-DFT calculated energies, percent contributions from dominant one-electron excitations, and oscillator strengths for the major electronic transitions in $[Mn^{III}(OH)(dpaq)]^+$ and $[Mn^{III}(OH)(^{6Me}dpaq)]^+$.

Ligand	State	Energy (cm^{-1})	f_{osc}	Transition	%	Donor MO (%)	Acceptor MO (%)	Comment
dpaq	1	13000 (770 nm)	0.00124	117a→119a	91.0	Mn $3d_{x^2-y^2}$ (39.1)<br>N p_x (19.9)<br>N p_y (14.8)	Mn $3d_{z^2}$ (51.5)<br>O p_z (10.3)<br>N p_x (6.2)<br>N p_z (7.6)	*d-d* & LMCT
	2	19800 (505 nm)	0.00113	118a→119a	81.5	Mn $3d_{yz}$ (2.3)<br>O p_y (9.6)<br>N p_y (23.4)<br>C p_y (54.7)	Mn $3d_{z^2}$ (51.5)<br>O p_z (10.3)<br>N p_x (6.2)<br>N p_z (7.6)	LMCT
	4	20600 (486 nm)	0.00238	114a→119a	38.9	Mn $3d_{xz}$ (11.7)<br>O p_x (43.4)<br>C p_y (23.6)<br>N p_x (5.2)	Mn $3d_{z^2}$ (51.5)<br>O p_z (10.3)<br>N p_x (6.2)<br>N p_z (7.6)	LMCT & *d-d*
				116a→119a	29.3	Mn $3d_{xz}$ (6.8)<br>O p_x (53.3)<br>O p_2 (56.9)	Mn $3d_{z^2}$ (51.5)<br>O p_z (10.3)<br>N p_x (6.2)<br>N p_z (7.6)	LMCT & *d-d*
^{6Me}dpaq	1	15900 (630 nm)	0.00051	125a→127a	86.1	Mn $3d_{x^2-y^2}$ (33.4)<br>N p_x (16.4)<br>N p_y (19.1)	Mn $3d_{z^2}$ (45.8)<br>O p_z (8.8)<br>N p_x (6.2)<br>N p_z (7.0)	*d-d* & LMCT
	3	20000 (498 nm)	0.00052	126a→127a	58.5	Mn $3d_{yz}$ (2.4)<br>N p_y (22.9)<br>O p_y (9.4)<br>C p_y (48.8)	Mn $3d_{z^2}$ (45.8)<br>O p_z (8.8)<br>N p_x (6.2)<br>N p_z (7.0)	LMCT
				126a→128a	15.7	Mn $3d_{yz}$ (2.4)<br>N p_y (22.9)<br>O p_y (9.4)<br>C p_y (48.8)	N p_y (15.3)<br>C p_y (67.2)	ILCT
	4	21300 (467 nm)	0.00345	120a→127a	10.1	Mn $3d_{xz}$ (3.0)<br>O p_x (12.2)<br>C p_y (64.9)	Mn $3d_{z^2}$ (45.8)<br>O p_z (8.8)<br>N p_x (6.2)<br>N p_z (7.0)	LMCT & *d-d*
				121a→127a	43.1	Mn $3d_{xz}$ (12.4)<br>O p_x (52.3)<br>C p_y (13.9)	Mn $3d_{z^2}$ (45.8)<br>O p_z (8.8)<br>N p_x (6.2)<br>N p_z (7.0)	LMCT & *d-d*
				124a→127a	27.0	Mn $3d_{xz}$ (5.3)<br>O p_x (53.1)<br>C p_x (6.3)<br>C p_z (8.5)	Mn $3d_{z^2}$ (45.8)<br>O p_z (8.8)<br>N p_x (6.2)<br>N p_z (7.0)	LMCT & *d-d*

Calculation of Thermodynamic Parameters

The pK_a value, reduction potential, and BDFE of [$Mn^{III}(OH)(^{6Me}dpaq)$](OTf) were determined following an approach we previously employed for Mn^{III}-hydroxo complexes.[1] This procedure is based on principles employed in the work of Solis and Hammes-Schiffer.[2] This technique relies on the use of a reference complex (similar to the complex of study), for which experimental pK_a and reduction potential values are known. We utilized the [$Mn^{III}(OH_2)(dpaq)]^{2+}$ complex as our reference, with pK_a = 6.8 and $E_{1/2}$ = 0.65 (versus Fc^+/Fc).[3] The calculated pKa and $E_{1/2}$ parameters were determined using the optimized structures of the [$Mn^{III}(OH)(^{6Me}dpaq)]^+$, [$Mn^{II}(OH_2)(^{6Me}dpaq)]^+$, [$Mn^{II}(OH)(^{6Me}dpaq)$], and [$Mn^{III}(OH_2)(^{6Me}dpaq)]^{2+}$complexes. Single-point energy and analytical frequency calculations for these optimized structures provided the data for the determination of $\Delta\Delta G$ for the proton- and electron-transfer reactions between the reference compound and the compound of interest (Table S1). These $\Delta\Delta G$ values were then converted to pK_a and $E_{1/2}$ values relative to the reference complex (Table S2). From these values, the BDFEs for the [$Mn^{II}(OH_2)(^{6Me}dpaq)]^+$ complexes was calculated using the Bordwell equation.[4]

Table S3. DFT-Calculated ΔG values (in kcal/mol) for the addition of a proton or electron used in calculating the E_{red} and pKa values.

Ligand	$\Delta G(Mn^{III}(OH_2)$-$Mn^{III}(OH))$	$\Delta G(Mn^{II}(OH_2)$ - $Mn^{III}(OH_2))$	$\Delta G(Mn^{II}(OH)$ - $Mn^{III}(OH))$	$\Delta G(Mn^{II}(OH_2)$ - $Mn^{II}(OH))$
dpaq	-266.7	-109.4	-79.9	-296.2
6Medpaq	-266.4	-110.7	81.9	-295.2

Table S4. DFT-calculated thermodynamic parameters used to determine Mn^{II}-OH_2 BDFEs for the [$Mn^{II}(OH_2)(^{6Me}dpaq)]^+$ complex.

Ligand	pKa(Mn^{III}–OH_2)	E($Mn^{III/II}$–OH_2)	pKa(Mn^{II}–OH_2)	E($Mn^{III/II}$–OH)	BDFE
dpaq	6.8	0.65	29.3	-0.69	79.1
6Medpaq	6.6	0.71	28.6	-0.60	80.2

Table S5. Coordinates for the DFT optimized structure of $[Mn^{III}(OH)(dpaq)]^+$

	x	y	z
Mn	-0.14219165609036	-0.07253586590930	0.03607249738240
O	0.33122965488788	-1.20168526217487	-3.87229617381038
O	-0.19849237073471	-0.09656943160246	1.86376611350255
H	-1.07143935588853	-0.33399235757235	2.21202830594289
N	-0.74660079068655	1.90600332187026	-0.25519170390517
N	-0.07367099098497	0.00971768662552	-1.94109656174860
N	0.49902570255718	-2.16819791227168	-0.33473525618378
N	-2.06154811810668	-1.15962145752629	-0.14399246017459
N	2.06355247844104	0.04148873600204	0.09299462741103
C	-1.06408701816670	2.78969263609687	0.67596457829911
H	-0.99261608291609	2.45143625024986	1.71116599527579
C	-1.47216841924601	4.09853048131705	0.35111831754528
H	-1.72597014373794	4.79406615163105	1.15206916882673
C	-1.54174055117505	4.47116536795690	-0.97690634644377
H	-1.85452554376855	5.47952343220576	-1.25832033430262
C	-1.20424776960893	3.54132208662100	-1.99516424640734
C	-1.24311008470694	3.83163626758326	-3.38520008618476
H	-1.54824720293904	4.82628500693898	-3.71687814518010
C	-0.89252791977610	2.84886212218416	-4.28885452579482
H	-0.91842240184035	3.06452695568419	-5.36003668391427
C	-0.49475060841919	1.55279287888408	-3.87643593790228
H	-0.22682607705947	0.80480287910606	-4.61709996040394
C	-0.44281153255795	1.22597258917500	-2.52318705495967
C	-0.80329100964632	2.24044649886853	-1.57993259489325
C	0.28020121629306	-1.09407405226742	-2.64585501388067
C	0.67223727140655	-2.30805582891548	-1.80363972798089
H	1.72591941888893	-2.52389155739413	-2.03312696054286
H	0.09713229013950	-3.16913969218246	-2.17349812585978
C	-0.57227892626038	-3.03411677520719	0.19304539760490
H	-0.48654094066340	-3.03999654152999	1.28967193297595
H	-0.46008249410669	-4.07280213364442	-0.15482043089258
C	-1.93488472520184	-2.49544924835089	-0.16775823889411
C	-3.02093342803376	-3.31935302926983	-0.46054247491006
H	-2.89041743724205	-4.40249836019944	-0.48746865872848
C	-4.26246282396130	-2.73280048625363	-0.71566194050750
H	-5.12830177209073	-3.35675660327192	-0.94754215356754
C	-4.37963123313547	-1.34161386380974	-0.67891486582871
H	-5.32960808468429	-0.84273767826421	-0.87656874705749
C	-3.24359145365833	-0.58854096352645	-0.39343982478705
H	-3.27680640627013	0.50260522246721	-0.36586519396218
C	1.76744457573486	-2.33913153612788	0.40041595537648
H	1.52620994391939	-2.40327132821974	1.47153941338759
H	2.27763772245356	-3.27232813666299	0.11417501220687
C	2.67220707288147	-1.14965947041526	0.18954981062438
C	4.06165480899833	-1.25829745796042	0.13539694588957

H	4.53374719135460	-2.23991464637611	0.20317335641537
C	4.82298941239088	-0.09668055245048	-0.00828209839859
H	5.91276250232629	-0.15502194972326	-0.05488246915240
C	4.17398668232074	1.13664155004439	-0.10080299045284
H	4.73052016322734	2.06759088355517	-0.21903931884633
C	2.78253550324586	1.15798320779004	-0.05030498303693
H	2.22523276189734	2.09380696622294	-0.13046213917051

Table S6. Coordinates for the DFT optimized structure of $[Mn^{III}(OH)(^{6Me}dpaq)]^{+}$

	x	y	z
Mn	-0.02567476246197	-0.04459169029074	0.09967676049221
N	-1.97658679169226	0.48071393361650	-0.23448955959005
N	0.04117646579237	-2.37712738434784	-0.05675372548970
N	0.02390988620018	0.11438890384439	-1.86119582667103
O	-0.10295208514034	-0.14014504139624	1.92555675628101
O	1.41441794024280	0.03561908219863	-3.71112011905760
N	2.04742738182688	-0.49135744580290	-0.13896635387216
N	1.01768844183736	2.14142266563744	0.13868260506896
C	-2.92397189871559	0.62440534779709	0.67654105951924
C	-4.23523883992121	1.00259944981477	0.32868030342349
C	-4.54256881495845	1.23061730011237	-0.99893059648529
C	-3.54265780553904	1.08109311560620	-1.99568150066262
C	-3.75567916018994	1.29104295608044	-3.38495310266915
C	-2.70567350462418	1.11424054593534	-4.26402608975788
C	-1.41348520289158	0.72641062064980	-3.82813637663359
C	-1.16500025012785	0.51039398236158	-2.47587853854953
C	-2.24597735654061	0.69481025250400	-1.55915386605533
C	1.19773770827417	-0.09746916013351	-2.50712484192533
C	2.31850662121106	-0.60333084421137	-1.60276354506757
C	2.29740959911618	-1.76817779948695	0.56853766688011
C	1.29062458183852	-2.81275710370378	0.16827099930169
C	1.64033657156648	-4.15813853068180	0.06833557474556
C	0.64614676641376	-5.07782871798268	-0.26454970938781
C	-0.64881528145147	-4.61849225712816	-0.49497738454244
C	-0.92735863261562	-3.24944186837841	-0.39018018355329
C	2.83836881995795	0.59430208467451	0.48442462258636
C	2.34665812787011	1.95355609153614	0.06671528736203
C	3.22529051670481	2.96499987118492	-0.31215629781708
C	2.69886822508009	4.22613460367792	-0.60000751175311
C	1.32586981251519	4.42391617862421	-0.48915695331727
C	0.49766011630384	3.35371336998579	-0.11712982586957
C	-2.30381482939073	-2.71541353373950	-0.64951736217012
C	-0.98605839688791	3.53697463354196	0.00451003070375
H	0.05444595818788	0.71848967417961	2.34749415167464
H	-2.63551178529413	0.43509053334714	1.71151516755635
H	-4.98514563438154	1.11017686656478	1.11331495860291
H	-5.55189999967400	1.52580708163302	-1.29512895194754

H	-4.74617983923081	1.58762921721358	-3.73565238115250
H	-2.86403130662675	1.27315269359910	-5.33350761850459
H	-0.61076427870865	0.59341290280540	-4.54857562759415
H	2.47225325537963	-1.66056252638514	-1.86102423209632
H	3.24873896676434	-0.07833230745454	-1.86023117213415
H	2.19745512028514	-1.57055201437627	1.64537478162750
H	3.32282179934135	-2.12052523816664	0.38017206782263
H	2.67062091819616	-4.47026187399267	0.24631850300523
H	0.88086836635426	-6.14109012147116	-0.35205554717658
H	-1.44878621760418	-5.30973237973225	-0.76443458164830
H	3.90612557057667	0.47262405107894	0.24913414724098
H	2.72045444206872	0.50100814834383	1.57343441457034
H	4.29644098981026	2.76661879715429	-0.37291003826189
H	3.35502433537005	5.04557203081868	-0.90187347443179
H	0.88223444582665	5.40032428720937	-0.69037907683012
H	-2.69053548308173	-2.18316718667333	0.23177823394038
H	-2.99966873299650	-3.52786891719292	-0.89600164349648
H	-2.29412645171946	-2.00735250810862	-1.49104296665313
H	-1.50211199433299	3.13418306550476	-0.88023976541721
H	-1.24250118117270	4.60238579830533	0.07813151763140
H	-1.37688723294067	3.01699831369557	0.88909673820561
O	10.58863310015237	5.15652843088464	10.60319538910858

Table S7. Coordinates for the DFT optimized structure of $[Mn^{III}(OH_2)(^{6Me}dpaq)]^{2+}$

	x	y	z
Mn	0.01905221089840	-0.06571896570329	-0.04390103523411
N	-1.90543687505187	0.46032219491222	-0.35119327308200
N	0.02301385132093	-2.35994599040243	0.03469327251355
N	0.09986073694361	0.11886427718180	-1.94819680981538
O	-0.10960119647736	-0.10046076716661	1.97757812265429
O	1.51640923880734	0.07699727380871	-3.76856656012384
N	2.05658733117237	-0.51788097756336	-0.21355266665805
N	0.96097444498162	2.08585692695214	0.19955046501092
C	-2.86362543024227	0.58546003687148	0.55662522476488
C	-4.16315633022645	0.98918021687070	0.20554542164452
C	-4.45423030512674	1.26694923645663	-1.11783039250579
C	-3.44621555950808	1.14099606408393	-2.10756855278146
C	-3.63422553276726	1.40286738279714	-3.49165886358498
C	-2.57423596058772	1.24522503955473	-4.36139907646225
C	-1.29249755194070	0.82532940023660	-3.92177548225852
C	-1.07624337561538	0.55956332152775	-2.57578765869113
C	-2.16055029439843	0.72402693182999	-1.67179639827532
C	1.29357525404584	-0.10399807362547	-2.58097209613010
C	2.35411095615065	-0.69340231757978	-1.66771883835359
C	2.30993622937769	-1.77312751088922	0.54473318054682
C	1.28050182070924	-2.81261967292524	0.20339148616312
C	1.61153009666001	-4.15877287636816	0.09564981031047

C	0.59279324353295	-5.06933024591417	-0.18725650302599
C	-0.70531092204058	-4.59741037372563	-0.36040113812729
C	-0.97011406141887	-3.22648968041608	-0.25075602521090
C	2.84468884378269	0.60004029587419	0.37588482205409
C	2.28531486571709	1.93847760016633	-0.01091148709077
C	3.08852271293045	2.96750220666052	-0.48490919783342
C	2.49544007211825	4.21052787959338	-0.72527541519589
C	1.14013332038155	4.37203721381379	-0.46223299396439
C	0.38392470815800	3.28519338809991	0.00463479553688
C	-2.35443782068590	-2.69224848893504	-0.45362632120742
C	-1.07654591016836	3.44657560084905	0.30183830313007
H	0.20482844249446	0.63691073738748	2.53554873139540
H	-2.60243403657797	0.36643335192787	1.59228888173554
H	-4.91854094471118	1.07892907877016	0.98659218222664
H	-5.45689712226040	1.58452133330122	-1.41243231083487
H	-4.61514493949178	1.72535762660264	-3.84556224269470
H	-2.71341568073531	1.44512656755352	-5.42617834450195
H	-0.48261937208478	0.70932811438638	-4.63667797461669
H	2.39926067600902	-1.76738158210730	-1.89652922096446
H	3.33707510295962	-0.26924619432963	-1.90979877290264
H	2.25559714412567	-1.53493188800410	1.61652787514009
H	3.32701537718253	-2.13529061380452	0.33938375272860
H	2.64563333288398	-4.47940970104758	0.22804001471516
H	0.81220617433540	-6.13505014139410	-0.28099529906683
H	-1.52320705545054	-5.28132293123752	-0.59058872897515
H	3.89898566627230	0.50207351764696	0.08497220905028
H	2.78838432242641	0.50193712759534	1.46885706529982
H	4.15320577894661	2.80059676808245	-0.65304200394901
H	3.09086157744228	5.04466975972934	-1.10239254445936
H	0.65129037692180	5.33560631384962	-0.61338522297117
H	-2.69984178401651	-2.14466287529752	0.43482337602732
H	-3.06078943430370	-3.50915219211310	-0.64763373720013
H	-2.38743246848147	-2.00677525114875	-1.31320453919100
H	-1.68913073878862	3.15997242941179	-0.56699072361551
H	-1.30787251918667	4.49699937387504	0.52454812920924
H	-1.37788101231032	2.83222325574168	1.15945762606172
H	-0.15220681003347	-0.90819134730390	2.52151500363678

Table S8. Coordinates for the DFT optimized structure of [Mn^{II}(OH)(^{6Me}dpaq)]

	x	y	z
Mn	-0.14453527641658	-0.21809867116731	0.33083530573059
N	-2.18640315758702	0.45611099652977	-0.34604530582938
N	0.17511498039014	-2.53677386733417	0.12303321329971
N	0.01504047582641	0.16438358190920	-1.85208840772466
O	-0.39111886265709	-0.25673592007281	2.29855478597502
O	1.52343296559108	0.26886092195927	-3.63011068954887

N	2.12251111100167	-0.58581941009260	-0.14729017547205
N	0.99063436038475	2.08349653157401	0.32483430689659
C	-3.22253572090189	0.57888260037317	0.45922351725368
C	-4.45302654919837	1.12609787646094	0.03584451030318
C	-4.57100603060795	1.55218998005115	-1.27092040259701
C	-3.46862462588083	1.43516297249835	-2.15962339074966
C	-3.51463324394159	1.86286195431683	-3.51346536574560
C	-2.38799993566638	1.72028835092733	-4.29899858722373
C	-1.18918341896715	1.16372650746433	-3.79618667042738
C	-1.08481149002035	0.72338402356329	-2.47063894963060
C	-2.26370753162490	0.86549008588611	-1.64073856310881
C	1.19860305312467	-0.00677550326816	-2.45984510795986
C	2.25857600991614	-0.72184321779031	-1.60672798989620
C	2.45465794665088	-1.82232454172702	0.56946021995645
C	1.45530932608231	-2.91370481910806	0.26156759932486
C	1.84785683843290	-4.24798267423000	0.14008473939286
C	0.87192241167201	-5.20937597081777	-0.12401022931784
C	-0.45496908365570	-4.80655148792737	-0.26867935258450
C	-0.77517335748016	-3.44847102819169	-0.14263963580456
C	2.87035114184523	0.56754097145170	0.37422524858223
C	2.25691452533762	1.87157939613578	-0.07551884808670
C	2.95563316297708	2.79342250773102	-0.85126173665054
C	2.31137053233895	3.98087151597972	-1.21576818404282
C	1.00922583756835	4.20177751301298	-0.78119004135263
C	0.36964589152627	3.22324254905356	0.00063257787792
C	-2.17896821085509	-2.94463124974748	-0.30574818305647
C	-1.03512545497397	3.42748010812856	0.49416286755490
H	-0.24962447959370	0.61002450871254	2.69789677520038
H	-3.07386830752992	0.24020901489475	1.48817335115822
H	-5.28269973837482	1.20856384007592	0.74042390876463
H	-5.50526592542264	1.98573756283493	-1.63742841856074
H	-4.43564853654990	2.29794372581576	-3.90852963767037
H	-2.41215193029157	2.04697944374691	-5.34262310935231
H	-0.31902583637451	1.07117363649805	-4.44042807402450
H	2.17622852781139	-1.78726056554148	-1.86939901428450
H	3.25487758953495	-0.39227880995120	-1.94588852759769
H	2.41245392434006	-1.60303365340666	1.64796366601588
H	3.47730534585455	-2.17503795663472	0.34434108493839
H	2.89964758278432	-4.52038918917312	0.24445222291521
H	1.14521226048049	-6.26231024136035	-0.22686208645267
H	-1.24140436855516	-5.53122897340620	-0.48665001044225
H	3.93333040578182	0.52374639822630	0.07647335207905
H	2.82305941419478	0.52404203198143	1.47192693460885
H	3.98009124428446	2.58514410824562	-1.16471916430378
H	2.82582381938736	4.72487114816642	-1.82899420870968
H	0.47988564331657	5.12212985645315	-1.03707198436248
H	-2.50807727125867	-2.41361030346307	0.60073581696996
H	-2.87987050640727	-3.76620328223198	-0.50565731032080

H	-2.23439750753533	-2.22675860431316	-1.13862575561044
H	-1.75921574200334	3.29500927450546	-0.32556803666326
H	-1.16959889621565	4.44924251288694	0.88183633806399
H	-1.27612733188964	2.70664393290545	1.28504881230294

Table S9. Coordinates for the DFT optimized structure of $[Mn^{II}(OH_2)(^{6Me}dpaq)]^+$

	x	y	z
Mn	-0.08661871644147	-0.21508642637688	0.04734994738621
N	-2.11212767884189	0.45037198880849	-0.54063779190015
N	0.14962414805521	-2.47770646703602	0.22435505895942
N	0.08888994846283	0.15100749797552	-2.05419806464004
O	-0.39420044915458	-0.15370462696922	2.26618661362503
O	1.65199213094165	0.31967265715080	-3.77523208164309
N	2.15094743621471	-0.61038855656012	-0.30682126431120
N	0.93064107065611	1.95054265430664	0.33362690470620
C	-3.16473721775327	0.55755712307160	0.24997454716490
C	-4.39095010523096	1.10135875045559	-0.18146871433807
C	-4.49436717516686	1.54640352060032	-1.48306011434974
C	-3.38168871580288	1.44521714092376	-2.35900621585943
C	-3.41838581523354	1.88688557487328	-3.70857488624917
C	-2.28902665214431	1.75420952933949	-4.49050774377346
C	-1.09432282676681	1.18980225429836	-3.98592816704111
C	-1.00388633857848	0.73559082604377	-2.66725265464412
C	-2.17993362096779	0.87329752175337	-1.83747306373235
C	1.29162731204479	-0.00895376678268	-2.63462362077018
C	2.29579857657277	-0.78333714942478	-1.76754112566622
C	2.48998987259291	-1.83742745266491	0.42878187635607
C	1.42953430868659	-2.89559621509961	0.24325772994169
C	1.76616972969204	-4.24381562804048	0.13652153960051
C	0.74122404927956	-5.18335226816243	0.02413757605252
C	-0.58066439670238	-4.74393548230782	0.01786808829855
C	-0.85078605775865	-3.37399387012779	0.11593495997706
C	2.90322088943428	0.55550220803138	0.18708236156637
C	2.19898788354105	1.85371665811193	-0.11784324606035
C	2.83400081796512	2.89877237738457	-0.77943887674928
C	2.13329515150347	4.09538689873367	-0.96423241423741
C	0.84443011284914	4.20490085179189	-0.45725628788644
C	0.26284374386583	3.10671956281892	0.19894872151367
C	-2.25416382332997	-2.84828415160039	0.09642524935665
C	-1.12059178944423	3.21351730033233	0.77226387608054
H	0.13088584714660	0.51617244826952	2.73636520423909
H	-3.04485340657330	0.21143540667783	1.27915950263475
H	-5.22810382796676	1.16769978722387	0.51511941327176
H	-5.42600363438968	1.98037240189840	-1.85480673219642
H	-4.33822495904265	2.32393077397079	-4.10337274000113
H	-2.30749488375242	2.09124768659602	-5.53046070842150
H	-0.22088341805881	1.09928330233865	-4.62647121043878

H	2.13869466175621	-1.84583326929581	-2.00745366183261
H	3.31694170696962	-0.52744237060529	-2.09198832970856
H	2.54388139602821	-1.58532464927408	1.49926227429784
H	3.47779101911773	-2.23390389838892	0.13978521020361
H	2.81508668335430	-4.54432403733434	0.14083491872210
H	0.97221856088501	-6.24755219848853	-0.06248329890819
H	-1.40722450624402	-5.45065782333966	-0.06957430608189
H	3.92837261451390	0.57026322208603	-0.21803481320429
H	2.97834785276226	0.46540295599665	1.28053317384160
H	3.85645605922132	2.77812753154451	-1.14056609272432
H	2.59735401074661	4.93406814270978	-1.48837592110530
H	0.27670542351963	5.13168744591380	-0.55948035126595
H	-2.44463159302794	-2.20811626514325	0.97117835973276
H	-2.98505265865250	-3.66724660034206	0.10447258359156
H	-2.42964494714168	-2.24106774073584	-0.80499676717797
H	-1.88066240688170	3.18747082056067	-0.02412635294262
H	-1.24179637492487	4.17249379215143	1.29881588969121
H	-1.32596171602652	2.39876862325393	1.47648505489285
H	-0.29682444237854	-0.97551880989668	2.77927586215692

References

1. Rice, D. B.; Munasinghe, A.; Grotemeyer, E. N.; Burr, A. D.; Day, V. W.; Jackson, T. A., Structure and Reactivity of (μ-Oxo)dimanganese(III,III) and Mononuclear Hydroxomanganese(III) Adducts Supported by Derivatives of an Amide-Containing Pentadentate Ligand. *Inorganic Chemistry* **2019,** *58* (1), 622-636.
2. Solis, B. H.; Hammes-Schiffer, S., Proton-Coupled Electron Transfer in Molecular Electrocatalysis: Theoretical Methods and Design Principles. *Inorganic Chemistry* **2014,** *53* (13), 6427-6443.
3. Sankaralingam, M.; Lee, Y.-M.; Karmalkar, D. G.; Nam, W.; Fukuzumi, S., A Mononuclear Non-heme Manganese(III)–Aqua Complex as a New Active Oxidant in Hydrogen Atom Transfer Reactions. *Journal of the American Chemical Society* **2018,** *140* (40), 12695-12699.
4. Bordwell, F. G.; Cheng, J.; Ji, G. Z.; Satish, A. V.; Zhang, X., Bond dissociation energies in DMSO related to the gas phase values. *Journal of the American Chemical Society* **1991,** *113* (26), 9790-9795.
